# Cryptic Diversity within the Major Trypanosomiasis Vector *Glossina fuscipes* Revealed by Molecular Markers

**DOI:** 10.1371/journal.pntd.0001266

**Published:** 2011-08-09

**Authors:** Naomi A. Dyer, Sophie Ravel, Kwang-Shik Choi, Alistair C. Darby, Sandrine Causse, Berisha Kapitano, Martin J. R. Hall, Keith Steen, Pascal Lutumba, Joules Madinga, Steve J. Torr, Loyce M. Okedi, Michael J. Lehane, Martin J. Donnelly

**Affiliations:** 1 Vector Group, Liverpool School of Tropical Medicine, Liverpool, United Kingdom; 2 Institut de Recherche pour le Développement (IRD), UMR 177 IRD-CIRAD, LRCT Campus International de Baillarguet, Montpellier, France; 3 School of Biological Sciences, University of Liverpool, Liverpool, United Kingdom; 4 Southern Rift Valley of Ethiopia Tsetse Eradication Project, Hawassa, Ethiopia; 5 Natural History Museum, London, United Kingdom; 6 Department of Tropical Medicine, School of Medicine, Kinshasa University, Kinshasa, Democratic Republic of Congo; 7 Agriculture, Health and Environment Group, National Resources Institute, University of Greenwich, Chatham Maritime, United Kingdom; 8 National Livestock Resources Research Institute, Tororo, Uganda; 9 Department of Microbiology, Colorado State University, Fort Collins, Colorado, United States of America; IRD/CIRDES, Burkina Faso

## Abstract

**Background:**

The tsetse fly *Glossina fuscipes s.l.* is responsible for the transmission of approximately 90% of cases of human African trypanosomiasis (HAT) or sleeping sickness. Three *G. fuscipes* subspecies have been described, primarily based upon subtle differences in the morphology of their genitalia. Here we describe a study conducted across the range of this important vector to determine whether molecular evidence generated from nuclear DNA (microsatellites and gene sequence information), mitochondrial DNA and symbiont DNA support the existence of these taxa as discrete taxonomic units.

**Principal Findings:**

The nuclear ribosomal Internal transcribed spacer 1 (*ITS1*) provided support for the three subspecies. However nuclear and mitochondrial sequence data did not support the monophyly of the morphological subspecies *G. f. fuscipes* or *G. f. quanzensis*. Instead, the most strongly supported monophyletic group was comprised of flies sampled from Ethiopia. Maternally inherited loci (mtDNA and symbiont) also suggested monophyly of a group from Lake Victoria basin and Tanzania, but this group was not supported by nuclear loci, suggesting different histories of these markers. Microsatellite data confirmed strong structuring across the range of *G. fuscipes s.l.*, and was useful for deriving the interrelationship of closely related populations.

**Conclusion/Significance:**

We propose that the morphological classification alone is not used to classify populations of *G. fuscipes* for control purposes. The Ethiopian population, which is scheduled to be the target of a sterile insect release (SIT) programme, was notably discrete. From a programmatic perspective this may be both positive, given that it may reflect limited migration into the area or negative if the high levels of differentiation are also reflected in reproductive isolation between this population and the flies to be used in the release programme.

## Author Summary

*Glossina fuscipes s.l.* tsetse flies are responsible for transmission of approximately 90% of the cases of Human African Typanosomiasis in Sub Saharan Africa. It was previously proposed on the basis of morphology that *G. fuscipes* is composed of three sub-species. Using genetic evidence from *G. fuscipes* nuclear, mitochondrial and symbiont DNA, we show that the morphological subspecies do not correspond well to genetic differences between the flies and morphologically similar flies may have arisen more than once in the evolution of this species. Instead, we found at least 5 main allopatrically distributed groups of *G. fuscipes* flies. The most genetically distinct group of flies originated from Ethiopia, where a sterile insect release programme is planned. Given that tsetse control often exploits species-specific behaviours there is a pressing need to establish the taxonomic status and ranges of these five groups. Moreover given that we were only able to perform limited sampling in many parts of the species distribution further groups within *G. fuscipes* are likely to be awaiting discovery.

## Introduction

Control of Human African Trypansomiasis (HAT) has largely been based upon the detection and treatment of human cases [1]. Anti-vector interventions, whilst hugely successful in reducing transmission of Animal African Trypanosomiasis (AAT), have rarely been implemented on a programmatic scale [2], [3]. Part of the explanation for the relative neglect of anti- vector interventions is that the majority of cases of HAT are transmitted by flies within the *Glossina palpalis* group which are less amenable to control using natural (insecticide-treated cattle) or artificial (traps and insecticide-treated targets) baits.

The recently launched Pan African Tsetse and Trypanosomiasis Eradication Campaign (PATTEC) has placed anti-vector interventions back on the agenda for HAT control. This initiative aims to identify, then eradicate discrete populations of tsetse flies. The programme is not reliant upon a single intervention but will take an integrated vector management (IVM) approach which tailors the interventions to the ecology and bionomics of the target species. Most interventions, such as aerial spraying, bait and trap methods and release of sterile irradiated-males (SIT), require a detailed understanding of the biology and population genetics of the target species. As discussed in two recent papers by Solano *et al* we are beginning to see population genetic data being used to target and tailor control strategies for some species within the *palpalis* group [4], [5]. However, for *Glossina fuscipes s.l.*, which is thought to vector approximately 90% of cases of HAT [6], very few molecular genetic studies have been conducted [7], [8], [9], .Consequently, at present, our understanding of the taxonomy and population structure of this “species” is too incomplete to fully inform intervention strategies.

A recent initiative to develop improved bait technologies for *G. fuscipes spp.* flies has revealed marked geographical differences in the response of flies to both odour and trap design. In Kenya *G. f. fuscipes* were unresponsive to any mammalian odour whilst in the Democratic Republic of Congo (DRC) *G. f. quanzensis* was responsive to pig odour [12]. Similarly, studies investigating the optimal orientation for the insecticide-treated, oblong cloth traps which are commonly used to control tsetse suggest that the visual responses of the putative sub-species may differ. *Glossina f. fuscipes* was equally attracted to traps in which the longest axis of the oblong was either parallel (horizontal) or orthogonal (vertical) to the ground [13] whereas *G.f. quanzensis* was apparently more attracted to horizontal oblongs (S. Torr, unpublished). If these, and other, differences in vision and odourant-mediated behaviour between the putative *fuscipes* subspecies reflect genetic differences population genetic approaches may be used to target interventions to populations with specific behaviours.

**Figure 1:**
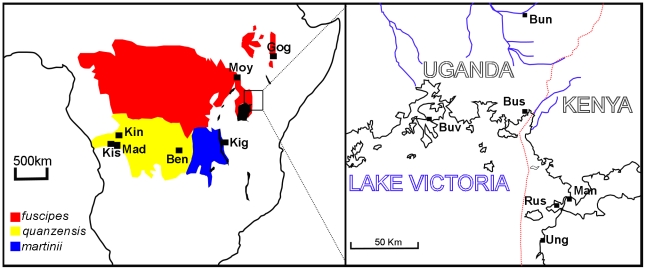
Distribution of the three putative subspecies of *G. fuscipes* and the sample sites of the 13 study populations. The right panel is a larger scale map of the Uganda/ Kenya region. Abbreviations on the left hand panel; Kin: Kinshasa; Mad: Madimba; Kis: Kisantu; Ben: Bena Tshibangu; Kig: Kigoma; Moy: Moyo; Gog: Gogara. Abbreviations on the right hand panel; Buv: Buvuma Island; Bus: Busime; Bun: Bunghazi; Rus: Rusinga Island; Man: Manga Island; Ung: Ungoye. The approximate distribution of the *G. f. fuscipes* is shaded red, *G. f. quanzensis* is shaded yellow and *G. f. martinii* is shaded blue.

*Glossina fuscipes s.l.* has an extensive distribution centralised on the Congo basin but also extending as far north as Ethiopia/Sudan and as far south as Angola (Figure 1). The sister group to *Glossina fuscipes* is the predominantly parapatric *Glossina palpalis* complex [9] whose species range lies largely to the west. Machado revised the systematics of the *palpalis* group, [14], and described three *G. fuscipes* subspecies on the basis of morphology. The first, *G. fuscipes fuscipes* inhabits the most humid, equatorial forest habitats across the northern part of the species range. The second subspecies, *G. f. martinii*, inhabits the south Eastern part of the range, around Lake Tanganyika, and in the drainage of river Lualaba from the south up to where it is joined by the Luama, and was described as the most tolerant of low humidity levels of the three subspecies. The third subspecies, *G. f. quanzensis*, is distributed in the south western part of the species range, in the drainages of the tributaries joining the Congo River south of Mushie. Machado asserted that the habitat of *G. f. quanzensis* is intermediate in character between *fuscipes* and *martinii*. Whether the present distributions are limited by the tolerance of the flies to different humidity levels is unknown, since only *G. f. fuscipes* has been empirically tested for desiccation tolerance [15]. The three subspecies are thought to have contiguous, non overlapping distributions. Machado concluded that the three *fuscipes* subspecies are probably the result of vicariant (allopatric) speciation events.

From the work of Vanderplank there is evidence for barriers to mating between some of the subspecies [16]. *Glossina fuscipes fuscipes* (then called *palpalis fuscipes*) from Uganda were reciprocally crossed with *G. fuscipes martinii* from Zambia [16]. In the female *G.f. fuscipes*×male *G. f. martinii* the superior claspers of the male genitalia punctured the female abdomen leading to death of the female. The reciprocal cross showed partially sterility, with approximately10 times fewer pupae produced than in intraspecific crosses. The area the subspecies inhabit has long been problematic to sample due to a combination of physical and socio-political difficulties and hence classical approaches of crossing different putative species are scant. In this paper by collecting samples over a wide geographical range and using molecular genetic approaches we attempt to determine whether the subspecies of *G. fuscipes sensu* Machado [14] are supported or if there is evidence for alternative genetic stratification within *G. fuscipes*. Given that methods of tsetse control often exploit species-specific behaviours there is a pressing need to establish the taxonomic status and ranges of the taxa within *G. fuscipes s.l.*

## Materials and Methods

### Specimen collection and identification

*G. fuscipes* were collected using biconical traps or pyramidal traps [17], [18] at the locations and dates shown in table 1 and figure 1. After preliminary morphological identification in the field, flies were stored in either acetone or 90% ethanol. In the laboratory samples were assigned to the three morphological subspecies proposed by Machado [14] using the identification key of Jordan [19].

**Table 1:** Summary of *G. fuscipes s.l.* material collection locations.

Town/Region	Country	Morphological Identification	Coordinates North/South	Coordinates East/West	Date collected	Sample size^1^
Kinshasa (Kin)	DRC	*quanzensis*	4°28′S	15°16′E	March 2007 and December 2007	N = 43
Madimba (Mad)	DRC	*quanzensis*	4°59′S	15°6′E	October 2007	N = 29
Kisantu (Kis)	DRC	*quanzensis*	5°8′S	15°5′E	Novermber 2007	N = 11
Bena Tshibangu (Ben)	DRC	*quanzensis*	6°11′S^2^	23°38′E^2^	September 2009	N = 38
Gogara ,Deme and Kulano Rivers (Gog)	Ethiopia	*fuscipes*	6°34′N	37°33′E	November 2007	N = 30
Moyo (Moy)	Uganda	*fuscipes*	3°39′N	31°43′E	May 2009	N = 40
Busime (Bus)	Uganda	*fuscipes*	0°14′N	33°57′E	September 2007	N = 23
Bunghazi (Bun)	Uganda	*fuscipes*	0°56′N	33°58′E	September 2007	N = 30
Buvuma Island (Buv)	Uganda	*fuscipes*	0°14′N	33°16′E	May 2007	N = 50
Ungoye (Ung)	Kenya	*fuscipes*	0°36′S	34° 5′E	September 2007	N = 35
Manga Island (Man)	Kenya	*fuscipes*	0°21′S	34°15′E	August 2007	N = 35
Rusinga Island (Rus)	Kenya	*fuscipes*	0°21′S	34°13′E	September 2007	N = 30
Kigoma (Kig)	Tanzania	*martinii*	4°52′S^2^	29°37′E^2^	April and October 2009	N = 38

1 Refers to the number of specimens screened in the microsatellite study.

2 Only approximate coordinates were available for the sampling site as only the name of the nearest village was recorded. *G. f. martinii* were captured in the Gombe Stream nature reserve near Kigoma. Both these locations are at least 640 km from the nearest neighbour.

### DNA extraction and amplification

For mitochondrial and nuclear sequence data, DNA was extracted from three legs per tsetse using a modified version of the Ballinger-Crabtree protocol [20], [21]. The same method was used to extract DNA from tsetse abdomens for the amplification of DNA from the tsetse symbiont *Wigglesworthia glossinidia*. The abdomen was used because *Wigglesworthia* is concentrated in a specialized organ, the bacteriome, on the tsetse midgut. Table S1 details which loci were examined in each specimen (Accession numbers HQ387026–HQ387133). The sequencing-based analyses were conducted at the Liverpool School of Tropical Medicine whilst microsatellite analyses were conducted at the Institut de Recherche pour le Développement. A Chelex method [22] was used to extract DNA from 3 legs of individuals used solely for microsatellite analysis.

An 850 bp fragment of the 3′ end of the *Glossina* mitochondrial *Cytochrome Oxidase 1* gene, a 764 bp fragment of the *Glossina* mitochondrial *NADPH dehydrogenase 2* gene, and a 618 bp fragment of *Glossina* ribosomal *internal transcribed spacer 1* (*ITS1*) were amplified as described previously [9] using primer pairs COI-CULR, TW-N1284- N2-J586 and Glossina ITS1for- GlossinaITS1rev respectively.

Putative *Glossina period* gene sequences were identified from genome reads produced by the Wellcome Trust Sanger Institute available from http://www.sanger.ac.uk/resources/downloads/vectors/glossina-morsitans-morsitans.html using tBLASTn with the *Drosophila melanogaster* period protein sequence (NP_525056) as a query seqeuence. *period* was selected as it is a single copy nuclear gene in *Drosophila* and other insects and has been previously used to study closely related taxa [23], [24]. tBLASTn hits to the *G. m. morsitans* genome (downloaded from Sanger website in February 2008, cut off probability 1×e^−20^) were assembled using CodonCode aligner (CodonCode corporation) together with the cDNA GMsg-3911 found using a description search of “*period*” in GeneDB (http://old.genedb.org/genedb/glossina) [25]. A possible intron-exon structure was inferred by comparison with *Drosophila* cDNA and genomic DNA, and the protein sequences of other insect *period* genes. Primers Perfor1 (GATTTCGTTCATCCCAAGGA) and Perrev1 (GAGGCTAAAGCCTGACAACG) were designed to amplify a fragment at the 5′ end of the putative *Glossina period* gene up to the highly conserved PAS domain (due to a gap in the blast hits, the precise length of the fragment was determined by PCR and sequencing to be 1026 bp). This fragment was initially amplified and sequenced from *G. m. morsitans* genomic DNA, and the same primers were subsequently used to amplify the same region from *G. fuscipes* genomic DNA. 25 µl reactions contained 1 µl template, 0.8 mM dNTP, 3 mM MgCl_2_, each primer at 0.5 µM and 0.08 µl (0.4 units) Kapa Taq polymerase. 30 amplification cycles of 94°C for 30 seconds, 60°C for 30 seconds and 72°C for 2 minutes were used. Primers and reaction conditions for the less variable 3′ region are given in the table S2 and Text S1.

*Wigglesworthia*. Genes for use as G. *fuscipes* genotyping markers were identified by comparative genomics between *W. glossinidia* - *G. brevipalpis* [26] and *W. glossinidia* - *G. m. morsitans* genomes (Serap Aksoy, pers. comm.). No *W. glossinidia* - *G. fuscipes* genome was available and differential gene loss in symbiont lineages was anticipated. To allow for the different gene content orthologous single copy genes were identified between *E. coli* (K1), *W. glossinidia* from *G. brevipalpis and G.m. morsitans*, using ORTHOMCL [27]. A total of 355 genes were found to be single copy and present in all three genomes. The gene orthologous groups where aligned using MUSCLE [28], only five genes showed high levels of divergence at the nucleotide level in genome regions with conserved synteny. Degenerate primers were designed to all regions, but the hypothetical protein *YcfW* was the only one which yielded amplicons of the selected size. The gene encoding the hypothetical protein, *YcfW*, was initially amplified using degenerate primers DG11F (5′-ACWTGGATKTYAAAATACGG-3′) and DG11R (5′-ACWCCTGAWAARTAYATTGG-3′) based upon sequences of *W. glossinidia* from *G. brevipalpis* (genome accession number: NC_004344) and *G.m. morsitans* (Serap Aksoy, *pers. comm*.). The degenerate primers amplified a 600 bp fragment from *G. fuscipes* derived material which was then sequenced and used to design specific primers for *G. fuscipes Wigglesworthia* (Gp11fusc_for 5′-GCGCTATTTTAATATCTTTTATTTTTG-3′; Gp11fusc_rev 5′-TGGATTWTCAGAACAAATDGTTAATC-3′). *YcfW* was amplified for 35 cycles of 94°C for 30 seconds, 58°C for 30 seconds and 72°C for 30 seconds from roughly 40 ng of template DNA extracted from either abdomen or the whole fly, with primers 0.5 µM each, MgCl_2_ 3 mM, dNTP, 0.8 mM. These primers amplified a 499 bp fragment and were also used for direct sequencing.

Sanger sequencing of PCR products was performed by Macrogen Korea using an ABI3730XL sequencer. PCR products were purified prior to sequencing using Sureclean (Bioline) according to the manufacturer's instructions.

### Microsatellites

All the individuals studied were genotyped using 5 microsatellite loci with dinucleotide repeats, using a LI-COR sequencer. All these microsatellite loci were originally isolated by Alan Robinson (Entomology Unit, Food and Agricultural Organization of the United Nations/International Atomic Energy Agency, Austria). GfA3, GfB8, and GfB101 were redesigned to produce smaller amplicons [29]. The PCR reactions were carried out in a thermocycler (MJ Research, Cambridge, UK) in 20 µl final volume using 10 µl of the diluted supernatant from the extraction step. After PCR amplification, allele bands were routinely resolved on a 4300 DNA Analysis System from LI-COR (Lincoln, NE) after migration in 96-lane reloadable (3×) 6.5% denaturing polyacrylamide gels. This method allows multiplexing by the use of two infrared dyes (IRDye™), separated by 100 nm (700 and 800 nm), and read by a two channel detection system that uses two separate lasers and detectors to eliminate errors due to fluorescence overlap. To determine the different allele sizes, a large panel of about 70 size markers was used. These size markers had been previously generated by cloning alleles from individual tsetse flies into pGEM-T Easy Vector (Promega Corporation, Madison, WI, USA), by sequencing the cloned alleles to determine their exact size. PCR products from these cloned alleles were run in the same acrylamide gel as the samples, allowing the allele size of the samples to be determined accurately [30]. Allele sizes were scored twice by two independent readers using the LI-COR Saga^GT^ genotyping software. Primers, repeat motifs, allele size ranges and the dye used are given in table S2.

### Sequence data analysis

Sequence data: An incongruence length difference (ILD)/partition homogeneity test [31] was performed in PAUP [32] to determine whether *Cytochrome oxidase 1* and *NADH dehydrogenase 2* sequences could be used together for estimating phylogenetic trees. No significant difference was detected between tree lengths of the *COI∶ND2* partition compared to random partitions of the same size, so subsequent tree inference was performed on the combined data set.

JModeltest [33], [34] was used to perform a hierarchical likelihood ratio tests on all markers to find which substitution model best describes their evolution. Using the Akaike information criterion (AIC), the Tamura Nei 1993 model [35] was specified for the *COI+ND2* dataset. This model was used to make maximum likelihood (ML) trees using PhyML online [36]. Neighbour joining trees were inferred using PAUP version 4.0 [32]. Other than specifying the substitution model and a gamma distribution of rates among sites, PAUP settings for distance trees were default except that base frequencies were determined empirically from the data, tree searching was heuristic with a random order of sequence addition repeated 10 times, and 2000 bootstrap replicates were performed. Using JModeltest, the “Transversion” model was specified for *YcfW* (AIC). This model is equivalent to the Generalised time-reversible (GTR) model but with only one transition rate. We therefore used the similar GTR model for neighbour joining trees in PAUP and for ML tree inference in PhyML.

Bayesian phylogenies of *COI+ND2, YcfW* and *Period* and *ITS1* were made using MrBayes [37], in each case the substitution model was selected using the Bayesian information criterion in JModeltest [33], [34]. For *COI+ND2* each gene was designated as a partition of the dataset. Both *YcfW* and *COI+ND2* were allowed 6 substitution rates with a gamma distribution of rates across sites and a proportion of invariable sites allowed. *Period* was permitted 2 substitution rates and no variation of rates among sites or invariable sites. *ITS1* was permitted one substitution rate and no variation of rates among sites or invariable sites. The rate prior was set to variable (dirichlet), with other priors left on default. Two runs of four chains were run for 2000000 generations, sampling every 100 generations. The first 100000 generations (1000 samples) were discarded as burn-in. Runs and burn-in of this length gave good convergence as assessed by examining plots of log probability against generation and observing that potential scale reduction factor for all parameters was close to 1. The analysis was repeated three times with different seeds for the random number generator.

For *period* full length sequences Jmodeltest specified the TPM3uf+G [38] substitution model under the Bayesian information criterion, and TIM3+G under the AIC. The TPM3uf+G model was used to infer a NJ tree using PAUP. The GTR model was used to infer a maximum likelihood phylogeny using PhyML online as this does not implement the TPM3uf or TIM3 models.

Molecular clock calculations on *COI* data were performed using divergence rate of 1.5% per million years appropriate for insect *COI* [39]. The assumption of uniform rates across the tree was not rejected by the two cluster test implemented in Lintre [40].

### Tests of monophyly

Hypotheses about monophyly were tested in a Bayesian framework by observing the frequency of particular groups being monophyletic in the posterior distribution of trees, which is the posterior probability of monophyly [41], [42]. The probability of monophyly of the three morphological subspecies (*fuscipes*, *quanzensis* and *martinii*) were tested, and we also tested the monophyly of Ethiopian *G. f. fuscipes* since these flies are geographically separated from other *G. fuscipes* by a discontinuity in their distribution, and the monophyly of Lake Victoria Basin (LVB) and Tanzanian specimens, since this seemed like a possible taxonomic unit in the *COI+ND2* tree. This was done by using PAUP [32] to filter the posterior distribution of trees excluding the burn-in (i.e. 19001 trees from each run) to find the trees which agree with the hypothesis of monophyly.

The Shimodaira and Hasegawa (SH) test [43] was also used to test the Maximum Likelihood tree topology under the constraints of the three morphological subspecies and the monophyly of Ethiopian *G. fuscipes*, estimating the bootstrap probabilities by bootstrap resampling the estimated log likelihoods of sites 1000 times (the RELL method) [44]. The monophyly of LVB+Tanzanian flies, a taxonomic unit noticed only after tree construction, was not tested using the SH test because hypotheses for this test should be *a priori* hypotheses, independent of the observed data [45].

### Microsatellite data analysis

Linkage disequilibrium (LD) between microsatellite loci was tested in each population using Genepop V [46]. A log likelihood ratio statistic (G test) was calculated for contingency tables of genotypes of each pair of loci in each sample. A global test for each pair of loci across all sample sites was also performed using Fisher's method. The Ethiopian sample sites were all considered as one due to their geographic proximity (<10 Km). Although the straight line distance was shorter between Manga and Rusinga islands (<5 km), they were considered separately because this distance is over open water. F_ST_ [47] was estimated with correction for null alleles, [48]. Null allele frequency was estimated using the expectation maximization algorithm of [49] using FreeNA [48], and was also estimated simultaneously with the inbreeding coefficient as described by Chybicki and Burczyk [50].

After re-coding positions in the matrix containing no data with a unique code, the ‘excluding null alleles’ (ENA) corrected and uncorrected genotype data was converted into PHYLIP format for further analysis with programmes within the PHYLIP package [51]. Recoding of missing data genotypes with a code unique for each locus was necessary to make the sum of allele frequencies 1. This makes the assumption that all missing data at a particular locus are the result of a single mutation that results in a null allele, which is an oversimplification. However, trees made using the original (non-recoded) dataset using populations [52] results in similar topology of the well supported clades, with only the poorly supported nodes changing. Allele frequencies were bootstrapped over loci using *seqboot*. The Cavalli-Svorza chord distance [53] was calculated using *gendist* and neighbour-joining trees made for each of the bootstrapped datasets using *neighbour*. An extended majority rule consensus tree of the bootstrap replicates was calculated using *consense*, the tree converted to an unrooted tree using *retree*, and branch lengths based on the non bootstrapped Cavalli-Svorza distance matrix were imposed on that tree topology using *fitch*, where negative branch lengths were not allowed.

Hierfstat [54] was used to test the contribution of hierarchical levels of population structure on departures from Hardy Weinberg equilibrium. Specifically, we aimed to test whether the morphological subspecific classification (F_subspecies/total_) accounts for a significant level of genetic differentiation once the geographical sampling is taken into account (F_cluster/subspecies_, F_sample site/cluster_ and F_individual/sample site_). Hierfstat tests the significance of higher levels of the hierarchy by permuting predefined units at a lower level between the bigger units defined by the higher level. Since *G. f. martinii* was only sampled from one site (Kigoma), this sample was removed from the dataset for Hierfstat analysis. Three levels of structure were considered above “individual”, which were sample site, geographic cluster (Kinshasa, Madimba and Kisantu were grouped into one cluster, Ungoye, Manga and Rusinga into another, and Busime and Buvuma into another, with the remaining sample sites classified individually. 1000 permutations were used to test the significance of F statistics at each level of the hierarchy, for all 5 autosomal loci and across all loci.

STRUCTURE 2.3.1 [55], [56] was used to infer population structure without prior information about sample locations. STRUCTURE assigns individuals to each of K clusters with different probabilities. STRUCTURE was run with K = 1 to K = 12, using 10 replicate runs for each value of K with sequential random seeds. A burn-in period of 12000 iterations and a subsequent 60000 iterations were used to estimate parameters. The admixture model was used, which assumes that a fraction of the genome of each individual can come from each of the K populations. Allele frequencies were allowed to be correlated between clusters, as each cluster is thought to have undergone genetic drift away from a common ancestral population. The optimal value of K was assessed using the DeltaK method of Evanno *et al* [57]. When the whole dataset was entered, K = 2 was the optimal number of clusters using this criterion, which is the uppermost level of hierarchical structure. We then aligned the results of the 10 runs with K = 2 using the full search algorithm implemented in CLUMPP [58]. The proportionate assignment of each individual output by CLUMPP was then used to assign each individual to one of three groups: 1. Assigned to cluster 1 with >90% probability, 2. Assigned to cluster 2 with >90% probability and 3. Assigned to neither cluster with >90% probability. Data from the third group was discarded for further analysis. Groups 1 and 2 were analysed separately in STRUCTURE as above, except that only K = 1−K = 10 was considered. For group 2, the greedy method, which selects the locally optimal solution at each stage in the hope of finding the global optimum, was used on CLUMPP since the full search algorithm took >5 minutes to run. STRUCTURE analysis was run with the original genotypes, and also with missing data genotypes replaced with a code unique for each locus.

## Results

### Sequence data

#### 1. Internal transcribed spacer 1 (ITS1)

The status of the morphological subspecies received initial support from ITS1 data. For ITS1 there were 12 variable positions in the 546 bp alignment. Of these, there were four fixed differences (Positions 27, 145, 170, 215 on the alignment, figure S1) between *G. f. martinii* and the other fuscipes subspecies. An additional two fixed differences (Positions 63, 328 on the alignment, figure S1) were observed between morphological *G. f. fuscipes*, and morphological *G.f. quanzensis* and *G. f. martinii*. These conserved differences were used to design what we must tentatively term a diagnostic PCR as specimen identifications were ambiguous. Details of the assay are provided in the supporting information section in the hope that other researchers may be able to improve the design when specimens become available from previously unsampled areas (Text S1, Figure S1 and Figure S2).

#### 2. Mitochondrial DNA

**Figure 2:**
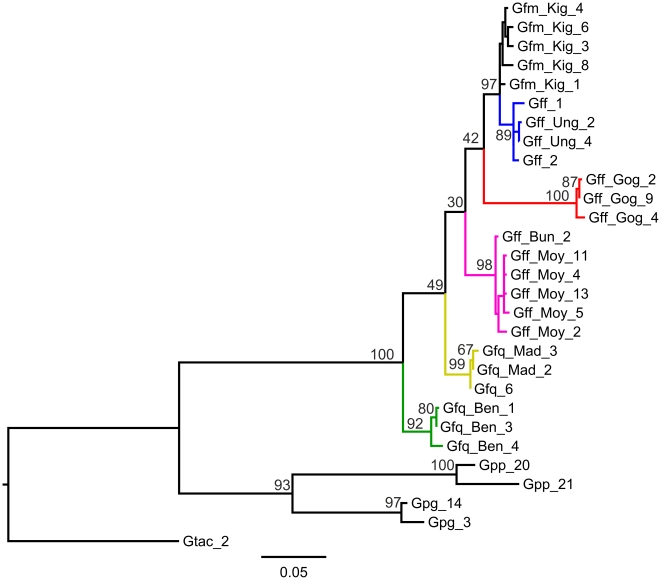
Maximum likelihood tree for mitochondrial genes (COI+ND2). Distances were calculated using the Tamura Nei (1993) substitution model. Node support is given as a percentage of bootstrap replicates (n = 1000). Branch colour reflects sample collection location; blue: Lake Victoria Basin; black: *G. f. martinii* from Tanzania; red: Ethiopia; pink: Mid/Northern Uganda; yellow: west DRC; green: Bena Tshibangu. The tree is rooted using specimens from three species within the *palpalis* group; Gpp: *Glossina palpalis palpalis*; Gpg: *Glossina palpalis gambiensis* and Gtac: *Glossina tachnioides*. See table S1 for key to specimen names.

*COI* and *ND2* sequences were concatenated, producing the gene tree shown in figure 2. Bayesian and distance trees were also made (figure S3). In contrast to the *ITS1* data there was no clear support for monophyly of the three morphological subgroups of Machado [14]. There was strong geographical clustering in the data set, *i.e.* no haplotypes originating from individuals sampled at a single geographic origin clustered with different sampling units. Lake Victoria basin samples (SE Uganda and Kenya) formed a well supported clade together with *G. f. martinii* from Tanzania, within which Lake Victoria Basin *G. f. fuscipes* is itself well supported. There was also strong support for the following clades; Ethiopian *G. f. fuscipes*; Mid plus north Ugandan *G. f. fuscipes*, *G. f. quanzensis* from Western DRC and *G. f. quanzensis* from Bena Tshibangu, central DRC. Whilst these five clades were well supported there was poor interclade resolution. The net divergence in *COI* at the deepest split in the ML and NJ trees (between East DRC and all other *G. fuscipes*) is 2.2% (SE 0.48%), which corresponds to a divergence time between 1.8 and 1.2 million years ago assuming a divergence rate of 1.5% per million years [39].

#### 3. *Wigglesworthia YcfW* gene

**Figure 3:**
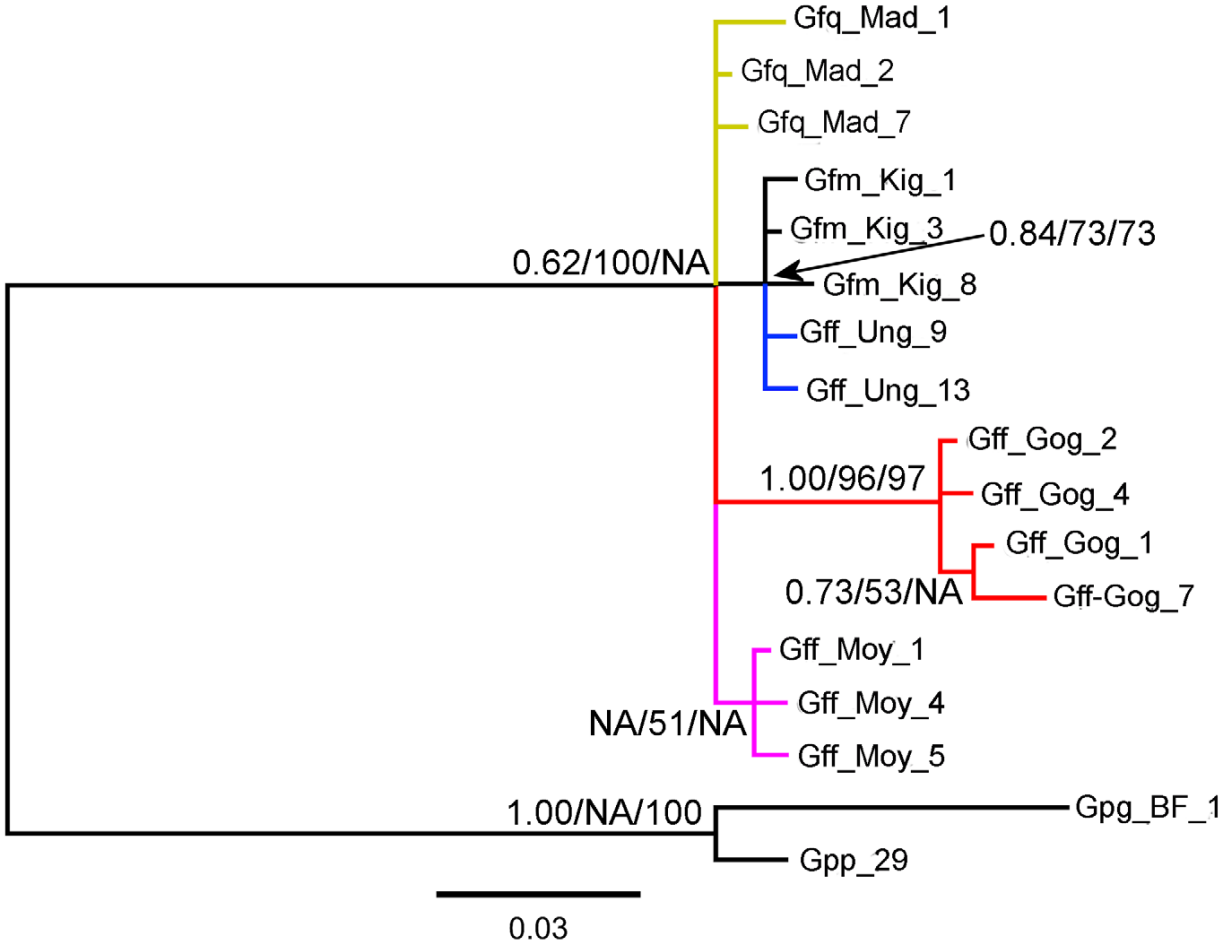
Bayesian gene tree for 434 bp alignment of *Wigglesworthia YcfW* gene. *G. p. palpalis* and *G. p. gambiensis* sequences were used to root the tree. Node support for shared nodes in the Maximum Likelihood (% of 1000 bootstrap replicates) and Neighbour Joining trees (% of 2000 bootstrap replicates) are shown after the posterior probability at each node. Branch colour reflects sample collection location; blue: Lake Victoria Basin; black: *G. f. martinii* from Tanzania; red: Ethiopia; pink: Mid/Northern Uganda; yellow: west DRC. See table S1 for key to specimen names.

The *Wigglesworthia* gene *YcfW* provided no clear support for the three morphological subgroups of Machado (Figure 3, figure S4). Again the best supported clade was the Ethiopian clade with marginal support for the Lake Victoria basin+*G. fuscipes martinii* clade.

#### 4. *Glossina Period* nuclear gene

Whilst preliminary analysis of period sequences across the genus Glossina suggested it was a good phylogenetic marker (figure S5) there were only 14 variable positions and consequently the gene trees were poorly resolved (figure S5).

### Tests of Monophyly

Bayesian tests of monophyly were performed for all sequence data sets (Table 2, figures S3, S4, S5). No marker supported the monophyly of *G. f. fuscipes* or *G. f. quanzensis*, although *ITS1* did provide weak support for the monophyly of *G. f. fuscipes* (P = 0.917). All the markers give support to the monophyly of *G. f. fuscipes* from Ethiopia. The monophyly of *G. f. martinii* was supported by the nuclear marker (*Period*), but neither of the maternally inherited markers. The hypothesis of the monophyly of flies inhabiting Lake Victoria basin down to Tanzania (LVB+*martinii*) is supported by mitochondrial DNA but rejected by the nuclear marker *period*, with *Wigglesworthia YcfW* being inconclusive. This contrast between nuclear and maternally inherited markers may reflect the repeated adaptive sweeps to which maternally inherited markers are prone which can result in dissociation between nucleotide diversity and population demography [59].

**Table 2:** Bayesian testing of monophyly: posterior probability of monophyly for each dataset.

Group	*COI + ND2* (mtDNA)	*COI + ND2* (mtDNA)	*YcfW* (*Wigglesworthia*)	*Period*	*ITS1*
N individuals	29 (all haplotypes)	16	14	16	14
Nst (rates)^a^	6	6	6	2	1
*G. f. fuscipes*	<2.63×10^−5^	<2.63×10^−5^	<2.63×10^−5^	<2.63×10^−5^	0.917
*G. f. martinii*	0.136	0.363	0.085	**0.999**	ND^d^
*G. f. quanzensis*	<2.63×10^−5^	0.207	0.082^c^	4.74×10^−4^	0.130
Ethiopia	**1.000**	**0.998**	**0.999**	**0.994**	ND
LVB + *martinii* b	**0.995**	**0.995**	0.864	<2.63×10^−5^	ND

Significant test results are shown in **bold** font. For the mtDNA data set the 16 individuals that were sequenced at all loci were analysed together with and then separately from the remaining specimens for which only mtDNA data were available. This allowed comparison of similar sized data sets for mitochondrial, symbiont and nuclear DNA data sets. The discrepancy between the sample size for *ITS1* and *Wigglesworthia YcfW* loci (n = 14) and the *Period* and *COI+ND2* loci (n = 16) is due to the lack of Bena Tschibangu and Buvuma genotypes from the former.

a Permitted number of nucleotide substitution rates.

b A combination of flies from Lake Victoria Basin (LVB) and *G.f. martinii*.

c Result is only for *quanzensis* samples from western DRC.

d not done.

The more conservative Shimodaira Hasegawa (SH) test of monophyly was performed on the same data sets. SH tests rejected monophyly for *G. f. quanzensis* only for the full COI+ND2 dataset (P = 0.003; n = 29), but when only the individuals genotyped at other loci were considered, monophyly could not be rejected (P = 0.729; n = 16). Monophyly was also rejected for *G. f. fuscipes* (P = 0.029) for the *YcfW* dataset (table S3). No hypothesis could be rejected with the *period* or *ITS1* data sets.

### Microsatellite data

No pair of loci showed significant LD after Bonferroni correction for multiple testing. Exact tests of heterozygote deficit [60] and highly variable F_IS_ values suggested the presence of null alleles (table S4). Estimated null allele frequencies and the population inbreeding coefficient (F) for each population are shown in table S5. For each locus estimated null allele frequency was >0.1 in at least one population. Once the data set was adjusted to account for the presence of null alleles, the population inbreeding coefficient was low (<0.1) for all populations except Ungoye and Bena Tshibangu.

If the three morphological subspecies, *sensu* Machado are valid phylogenetic entities, subspecific classification should account for a proportion of the genetic differentiation between populations. However, using a hierarchical analysis of F-statistics morphological subspecific classification was not found to be a major determinant of genetic differentiation among *G. fuscipes*. Subspecific classification was defined as one level of the hierarchy, and sampling site/clusters of sampling sites as other levels. It was not possible to test *G. f. martinii* using this method since this subspecies was only sampled at one site. With uncorrected genotypes, significant levels of genetic differentiation were accounted for by sample site (F_sample site/cluster_ = 0.020–0.113, P = 0.001–0.003) and sample site cluster (F_cluster/subspecies_ = 0.050–0.210, P = 0.001–0.003) but not by subspecific classification (F_subspecies/total_ = −0.045–0.056, P>0.05) at all 5 autosomal loci. P values and F statistics were similar for both uncorrected and ENA corrected [48] genotypes.

**Figure 4:**
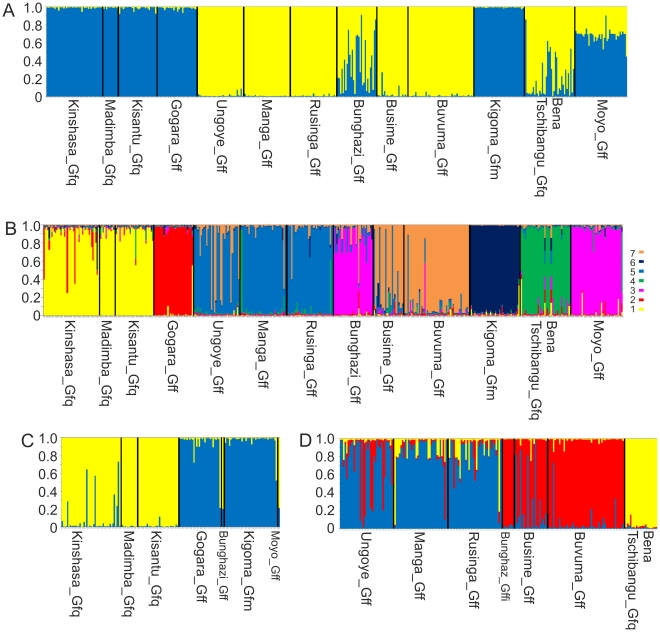
STRUCTURE analysis of microsatellite data. Output shown is for the original (not recoded) genotypes. The proportional assignment to a cluster is shown on the Y axis, with each narrow bar representing one individual. Sample sites are separated by black lines. Sample collection location names are given in full. The three letter abbreviated collection names used in figures 2 and 3 are the first three letters of the full name. The subspecies collected at each location is indicated by the suffix following the collection name. A. Proportional assignment of all genotyped individuals into k = 2 metapopulations 1 (yellow) and 2 (blue). B. Proportional assignment of all genotyped individuals into k = 7 metapopulations. C. Proportional assignment of individuals assigned with P>0.9 to metapopulation 1 into 2 clusters. D. Proportional assignment of individuals assigned with P>0.9 to metapopulation 2 into 3 clusters.

To test further the morphological subspecies hypothesis we used STRUCTURE software in the expectation that the genotypes would separate into three main clusters corresponding to the *martinii*, *quanzensis* and *fuscipes* subspecies within which there might be additional geographical sub-structuring. The optimal number of clusters based upon the DeltaK statistic was K = 2 [57], with a local peak at K = 7. Genotypes from Kinshasa, Madimba, Kisantu, Ethiopia, Kigoma fell into cluster 1, whereas those from Ungoye, Manga, Rusinga, Busime and Buvuma fell into cluster 2 (figure 4A). Bunghazi and Bena Tshibangu populations showed admixture between the two clusters, and Moyo was sometimes assigned to cluster 1 and sometimes to cluster 2. When K = 7, 5 of the clusters correspond to clades seen in the mitochondrial DNA trees, with the fifth mtDNA clade (Kenya and south Eastern Uganda) corresponding to the fifth (Kenya) and seventh clusters (SE Uganda) (figure 4B).

To further test the ability of the microsatellites to distinguish the morphological subspecies, genotypes assigned to cluster 1 or cluster 2 when K = 2, with probabilities greater than 0.9 were pooled into two separate data sets and the analysis re-run. For cluster 1, an optimal number of 2 sub clusters was found, which corresponded to i. West DRC (Kinshasa, Madimba and Kisantu), with ii. Ethiopia and western Tanzania (Kigoma) (figure 4C). Cluster 2 was separated into 3 subclusters, corresponding to i. Non admixed individuals from Bena Tshibangu, ii. eastern Lake Victoria Basin (Ungoye, Manga and Rusinge) and iii. northern Lake Victoria Basin (Busime and Buvuma) in the other, although Ungoye and Busime did show a moderate level of admixture (figure 4D). Thus the first level of clustering split *G. f. fuscipes* into two groups, one of which clustered together with *martinii* (Kigoma population), and the other of which corresponds to *fuscipes* living in the lake Victoria basin. *G.f.quanzensis* individuals (Western DRC and Bena Tshibangu) also failed to cluster together. At the next level of clustering, *martinii* was resolved as separate from *quanzensis*, but still grouped together with a *fuscipes* population (although at K = 3 or higher, *martinii* did cluster alone). When the analysis was run with null homozygotes recoded as homozygous for recessive alleles, the results were largely similar, except that Bena Tshibangu showed a higher level of admixture and therefore contributed very few individuals to the second runs on clusters 1 and 2. Cluster 1 split into an optimal (Max DeltaK) 3 clusters, which were i. West DRC, ii. Ethiopia and iii. Kigoma. STRUCTURE analysis does not support the hypothesis that the subspecies account for the deepest level of structuring amongst *fuscipes* populations.

Trees made using ENA corrected or uncorrected datasets were very similar in topology, only differing at nodes with <70% bootstrap support. As demonstrated by the low bootstrap values at internal nodes in this tree (figure S6), the phylogenetic relationships of widely geographically distributed *G. fuscipes* populations are not well resolved by this method. The only well supported clades are the Lake Victoria Basin (blue) and south west DRC (green). The distance of the morphologically similar Bena Tshibangu population from the other *quanzensis* flies is great, and they are not resolved as sister taxa in this tree.

## Discussion

There was not strong support for the three morphological subspecies proposed by Machado [14]. With the exception of *ITS1*, sequence data from both nuclear, mitochondrial and endosymbiont genomes rejected one or more of the morphological subspecies in tests of monophyly. Microsatellite data lends little support to the monophyly of *G. f. fuscipes*: in the STRUCTURE analysis the major subdivision between two clusters split *G. f. fuscipes* between these two clusters. The Hierfstat analysis showed that once the population differentiation due to sampling sites has been taken into account, subspecific identity does not contribute significantly to differentiation. Also, in the neighbour joining tree there was no clear separation into three clades according to Machado's subspecies. Both sequence and microsatellite data does however support Machado's statement that the subspecies are allopatrically distributed; no mixed taxonomic units or admixture between morphological subspecies is observed in any population.

However, microsatellite and mitochondrial DNA, and to a lesser extent *Wigglesworthia* DNA and single copy nuclear DNA did reveal strong support for marked genetic discontinuities within *G. fuscipes s.l.*

Taking the results from the various markers together, five clear sub divisions were observed:

- *G. f. quanzensis* populations from western DRC (supported by mtDNA, *period* and microsatellites).
- *G. f. martinii* samples from western Tanzania (supported by *period*, *ITS1*, *microsatellites*).
- *G. f. fuscipes* populations from central and northern Uganda (supported by mtDNA and possibly microsatellites). One of these populations is close to the type location collection site of Nimule, Sudan so it would be appropriate to term this group *G.fuscipes s.s.* In STRUCTURE analysis of microsatellite data with K = 2 however, Ugandan flies (Bunghazi and Moyo sites) did not fall neatly into either cluster. This fits well with the observations of Beadell *et al* [8] who proposed that the *fuscipes* currently inhabiting Uganda are the descendents of invaders that came in to the north of the Blue mountain range and the south of the Rwenzori mountain range. Beadell *et al* also observed the admixture of “northern and southern” lineages in Bunghazi, and proposed that the long separated lineages are now interbreeding in a zone of contact. In this study, Moyo was more frequently assigned to the cluster including Ethiopian and West DRC flies than the cluster including Kenyan and SE Ugandan flies, which would agree with it being dominated by “northern” genotypes.
- Lake Victoria basin *G. fuscipes* (supported by mtDNA and microsatellites), which we propose to provisionally identify as *G. fuscipes* type A.
- *G. f. fuscipes* populations from Ethiopia (supported by mtDNA, *YcfW*, *period* and microsatellites). We propose that this group is provisionally identified as *G. fuscipes* type B.

The status of the central DRC population from Bena Tshibangu was harder to resolve, despite being well supported by mtDNA, and forming a sister taxa to all other *fuscipes* in both the ML and NJ distance trees. The *Wigglesworthia YcfW* gene did not amplify from samples in this population using the same primers used to amplify the rest of the *G. fuscipes* specimens, which may suggest they harbour a very divergent sequence with mutations in the primer binding site. Judging by the number of locus specific non amplifications and heterozygote deficit, the microsatellite markers that were optimized on *G. f. fuscipes* populations from Uganda worked particularly poorly on the most divergent populations: *G. f. quanzensis* from central DRC, *G. f. fuscipes* from Ethiopia and *G. f. martinii*. Now that these samples are available, PCR primers to study these more divergent populations in more details could be designed for future studies.

One of the major difficulties in inferring the interrelationships of the *fuscipes* clades defined here was the lack of samples from the central and northern part of the species range, which lies mostly in DRC. The huge genetic distance between the central DRC population (Bena Tshibangu) and the Western populations (Kinshasa, Madimba and Kisantu) hints at large amounts of unexplored genetic structure within the morphological *quanzensis* types. Whether this marked genetic difference is associated with differing vectorial capacity is unknown but it is worth noting that there are marked differences in the epidemiology of HAT in the two locations. In central DRC HAT prevalence is very high whilst in western foci (Kinshasa and Bas Congo) HAT prevalence is very low [61], [62].

It also seems likely that the vast tracts of unstudied range of the *fuscipes* and *martinii* morphological types harbour yet more genetic structure. The *G. f. martinii* specimens used in this study were all collected from Kigoma, which likes on the Eastern shore of Lake Tanganyika, Tanzania. This is the eastern extreme of the range of the *martinii* type, and it is plausible that the *martinii* found to the West of Lake Tanganyika may be highly diverged from the specimens examined in this study. These unsampled populations will not change the conclusions of this study about the polyphyletic nature of the morphologically defined *G. f. fuscipes* and *G. f. quanzensis*. However, the dearth of samples from parts of the species range means that it would be premature to put forward a replacement for Machado's morphological subspecies theory.

We observed possible introgression between Lake Victoria Basin and Tanzanian flies: although we observed no shared mtDNA haplotypes between Tanzanian and LVB flies, the level of sequence divergence of as little as 2 substitutions (0.26%) between haplotypes is much less than would be expected from the high level of divergence observed at nuclear loci. This could indicate an introgression event that has later been obscured by genetic drift, or insufficient sampling of Tanzanian haplotypes. If the high similarity in maternally inherited but not biparentally inherited markers between the Lake Victoria Basin and *martinii* is due to introgression, it remains to be determined whether more westerly distributed *martinii* populations have been affected in the same way. Future studies of *G. fuscipes* genetic structure focussing in DRC, especially at the boundaries between the ranges of the morphological subspecies proposed by Machado will be essential to finally answer the question of *fuscipes* subspecies interrelationships. Since the mtDNA and *Wigglesworthia* DNA have been subject to at least one possible introgression event, nuclear microsatellites would be the most appropriate markers for this type of study, provided that sufficient numbers of unlinked polymorphic microsatellites can be found that amplify well in the more divergent and unstudied *fuscipes* populations.

Using molecular clock calculations on COI data, the most ancient divergence in the *fuscipes* group occurred between central DRC and all other *G. fuscipes* between 0.8 and 1.2 million years ago. This is much more recent than the split between *G. p. palpalis* and *G. p. gambiensis* which occurred between 4.2-2.2 Mya according to the same divergence rate of 1.5% per million years [63]. If behavioural divergence is correlated with genetic divergence within the *Glossina* then this relatively low level of differentiation may mean that similar control measures may be successful across the range so far studied. However, this conclusion must be viewed cautiously with respect to vector control, as it is already known that flies from Kinshasa and Kenya show different behaviours with respect to pig odour [12], and large tracts of *G. fuscipes* habitat in the DRC have not yet been studied. Therefore, it is advisable that any populations showing the same level of divergence as that observed between West DRC and Kenyan populations (Microsatellite null allele corrected F_ST_ = 0.26, 95% CIs = 0.17–0.36) may need to be tested separately for the efficacy of control measures. We would recommend that of the *G. fuscipes* populations studied so far, flies from Ethiopia and northern Uganda, Central DRC and morphological *G. f. martinii* (Tanzania) may require separate testing.

## Supporting Information

Figure S1 ***ITS1* sequence data alignment.** For specimen identities see Supplementary table 1. The first three rows are the primers used for the subspecies ‘diagnostic.’
(DOC)

Figure S2 **Agarose gel showing the provisional *Glossina fuscipes s.l.* form diagnostic PCR.** PCR products derived from template genomic DNA extracted from three individual *G. f. quanzensis*, three *G. f. martinii* and three *G. f. fuscipes* were separated next to a size marker by electrophoresis in agarose and stained with ethidium bromide. Product sizes are shown below the products.
(TIF)

Figure S3 **Bayesian and distance based neighbour-joining phylogenies based upon sequence data from the mtDNA *COI+ND2* genes.** Branch colour reflects sample collection location blue: Lake Victoria Basin; black; *G.f.martinii* from Tanzania; red: Ethiopia; pink: Mid/Northern Uganda; yellow: west DRC, green, Bena Tschibangu. A. Bayesian 29 taxa (full data set) used for Bayesian Phylogeny testing. Branch support is given as posterior probability. B. Bayesian phylogeny of the 16 taxa used for Bayesian Phylogeny testing. Branch support is given as posterior probability. C. Neighbour joining tree, based on Tamura and Nei (Tamura and Nei 1993. Molecular Biology and Evolution 10, pp 512–526) corrected distances. Branch support is shown as a percentage of 2000 bootstrap replicates.
(PDF)

Figure S4 **Bayesian phylogeny based upon sequence data from the *Wigglesworthia* locus, *YcfW* (14 taxa data set).** Used for Bayesian and Shimodaira-Hasegawa tests (Shimodaira and Hasegawa 1999. *Molecular Biology and Evolution* **16**, pp 1114–1116). Branch support is given as posterior probability. See Table S1 for key to specimen names.
(PDF)

Figure S5 **Bayesian, maximum likelihood and distance based neighbour-joining phylogenies based upon sequence data from the nDNA *Period* gene.** A. Gene tree for 2070 bp of period gene from selected taxa from genus Glossina. Node support for maximum likelihood and distance neighbour joining trees are given as a percentage of 1000 and 2000 bootstrap replicates respectively. B. Bayesian phylogeny for 5′ end of period gene (880 bp alignment) used for Bayesian and Shimodaira-Hasegawa tests (Shimodaira and Hasegawa 1999. Molecular Biology and Evolution 16, pp 1114–1116). Branch support is given as posterior probability. See Table S1 for key to specimen names.
(TIF)

Figure S6 **Neighbour-joining tree using Cavalli-Svorza distances for the microsatellite data set.** Cavalli-Svorza distances were calculated from ENA (Excluding Null Alleles: Chapuis and Estoup 2007. *Molecular Biology and Evolution* **24**, pp 621–631) corrected genotype data. Node support values are the proportion of 1000 bootstrap replicates over loci supporting that node. Branch colour reflects sample collection location blue: Lake Victoria Basin; black; *G. f. martinii* from Tanzania; red: Ethiopia; pink: Mid/Northern Uganda; green: DRC.
(TIF)

Table S1 Accession numbers for all sequence data.
(DOC)

Table S2 PCR conditions for amplification of the *Glossina Period* gene (A) and microsatellites (B).
(DOC)

Table S3 Results of Shimodaira-Hasegawa tests of monophyly.
(DOC)

Table S4 FIS per microsatellite locus and over all loci for each population.
(DOC)

Table S5 Estimated null allele frequencies at each microsatellite locus and population inbreeding coefficient F for each population.
(DOC)

Text S1 Methods for the *ITS1* based species “diagnostic” and period gene sequencing.
(DOC)
