# Supplementary material for: Cryptic Diversity within the Major Trypanosomiasis Vector Glossina fuscipes Revealed by Molecular Markers
Source: PLoS Negl Trop Dis. 2011 Aug 9;5(8):e1266. doi: 10.1371/journal.pntd.0001266 (PMC3153427; doi:10.1371/journal.pntd.0001266)
Supplement: Figure S1 — ITS1 sequence data alignment. For specimen identities see Supplementary table 1. The first three rows are the primers used for the subspecies ‘diagnostic.’ (DOC) [file pntd.0001266.s001.doc]

## Figure S1.

10 20 30 40 50 60 70 80 90 100

....|....|....|....|....|....|....|....|....|....|....|....|....|....|....|....|....|....|....|....|

**fus_quan_MIS** **----------------------------------------------------------------------------------------------------**

**mar_MIS**  **----------------------------------------------------------------------------------------------------**

**mar_quan_MIS** **----------------------------------------------------------------------------------------------------**

**Gfq_1**  **GTGATTATCAATTTTTGTTTATATATTTTATTTTGTCAATTAAATTCATATTTATTGAAAAACATTTTCAATAAATTTTTTTTTGTTATATGTACATTTT**

**Gfq_6**  **GTGATTATCAATTTTTGTTTATATATTTTATTTTGTCAATTAAATTCATATTTATTGAAAAACATTTTCAATAAATTTTTTTTTGTTATATGTACATTTT**

**Gfq_7**  **GTGATTATCAATTTTTGTTTATATATTTTATTTTGTCAATTAAATTCATATTTATTGAAAAACATTTTCAATAAATTTTTTTTTGTTATATGTACATTTT**

**Gfq_Mad_1**  **GTGATTATCAATTTTTGTTTATATATTTTATTTTGTCAATTAAATTCATATTTATTGAAAAACATTTTCAATAAATTTTTTTTTGTTATATGTACATTTT**

**Gfq_Mad_2**  **GTGATTATCAATTTTTGTTTATATATTTTATTTTGTCAATTAAATTCATATTTATTGAAAAACATTTTCAATAAATTTTTTTTTGTTATATGTACATTTT**

**Gfq_Mad_3**  **GTGATTATCAATTTTTGTTTATATATTTTATTTTGTCAATTAAATTCATATTTATTGAAAAACATTTTCAATAAATTTTTTTTTGTTATATGTACATTTT**

**Gfq_Ben_2**  **GTGATTATCAATTTTTGTTTATATATTTTATTTTGTCAATTAAATTCATATTTATTGAAAAACATTTTCAATAAATTTTTTTTTGTTATATGTACATTTT**

**Gfq_Ben_3**  **GTGATTATCAATTTTTGTTTATATATTTTATTTTGTCAATTAAATTCATATTTATTGAAAAACATTTTCAATAAATTTTTTTTTGTTATATGTACATTTT**

**Gfq_Ben_4**  **GTGATTATCAATTTTTGTTTATATATTTTATTTTGTCAATTAAATTCATATTTATTGAAAAACATTTTCAATAAATTTTTTTTTGTTATATGTACATTTT**

**Gfq_Ben_5**  **GTGATTATCAATTTTTGTTTATATATTTTATTTTGTCAATTAAATTCATATTTATTGAAAAACATTTTCAATAAATTTTTTTTTGTTATATGTACATTTT**

**Gff_1**  **GTGATTATCAATTTTTGTTTATATATTTTATTTTGTCAATTAAATTCATATTTATTGAAAAATATTTTCAATAAATTTTTTTTTGTTATATGTACATTTT**

**Gff_Buv_5**  **GTGATTATCAATTTTTGTTTATATATTTTATTTTGTCAATTAAATTCATATTTATTGAAAAATATTTTCAATAAATTTTTTTTTGTTATATGTACATTTT**

**Gff_10**  **GTGATTATCAATTTTTGTTTATATATTTTATTTTGTCAATTAAATTCATATTTATTGAAAAATATTTTCAATAAATTTTTTTTTGTTATATGTACATTTT**

**Gff_11**  **GTGATTATCAATTTTTGTTTATATATTTTATTTTGTCAATTAAATTCATATTTATTGAAAAATATTTTCAATAAATTTTTTTTTGTTATATGTACATTTT**

**Gff_12**  **GTGATTATCAATTTTTGTTTATATATTTTATTTTGTCAATTAAATTCATATTTATTGAAAAATATTTTCAATAAATTTTTTTTTGTTATATGTACATTTT**

**Gff_7**  **GTGATTATCAATTTTTGTTTATATATTTTATTTTGTCAATTAAATTCATATTTATTGAAAAATATTTTCAATAAATTTTTTTTTGTTATATGTACATTTT**

**Gff_8**  **GTGATTATCAATTTTTGTTTATATATTTTATTTTGTCAATTAAATTCATATTTATTGAAAAATATTTTCAATAAATTTTTTTTTGTTATATGTACATTTT**

**Gff_Gog_1**  **GTGATTATCAATTTTTGTTTATATATTTTATTTTGTCAATTAAATTCATATTTATTGAAAAATATTTTCAATAAATTTTTTTTTGTTATATGTACATTTT**

**Gff_Gog_2**  **GTGATTATCAATTTTTGTTTATATATTTTATTTTGTCAATTAAATTCATATTTATTGAAAAATATTTTCAATAAATTTTTTTTTGTTATATGTACATTTT**

**Gff_Kul_1**  **GTGATTATCAATTTTTGTTTATATATTTTATTTTGTCAATTAAATTCATATTTATTGAAAAATATTTTCAATAAATTTTTTTTTGTTATATGTACATTTT**

**Gff_Gog_9**  **GTGATTATCAATTTTTGTTTATATATTTTATTTTGTCAATTAAATTCATATTTATTGAAAAATATTTTCAATAAATTTTTTTTTGTTATATGTACATTTT**

**Gff_Moy_1**  **GTGATTATCAATTTTTGTTTATATATTTTATTTTGTCAATTAAATTCATATTTATTGAAAAATATTTTCAATAAATTTTTTTTTGTTATATGTACATTTT**

**Gff_Moy_2**  **GTGATTATCAATTTTTGTTTATATATTTTATTTTGTCAATTAAATTCATATTTATTGAAAAATATTTTCAATAAATTTTTTTTTGTTATATGTACATTTT**

**Gff_Moy_4**  **GTGATTATCAATTTTTGTTTATATATTTTATTTTGTCAATTAAATTCATATTTATTGAAAAATATTTTCAATAAATTTTTTTTTGTTATATGTACATTTT**

**Gfm_Kig_1**  **GTGATTATCAATTTTTGTTTATATATATTATTTTGTCAATTAAATTCATATTTATTGAAAAACATTTTCAATAAATTTTTTTTTGTTATATGTACATTTT**

**Gfm_Kig_3**  **GTGATTATCAATTTTTGTTTATATATATTATTTTGTCAATTAAATTCATATTTATTGAAAAACATTTTCAATAAATTTTTTTTTGTTATATGTACATTTT**

**Gfm_Kig_4**  **GTGATTATCAATTTTTGTTTATATATATTATTTTGTCAATTAAATTCATATTTATTGAAAAACATTTTCAATAAATTTTTTTTTGTTATATGTACATTTT**

**Gfm_Kig_6**  **GTGATTATCAATTTTTGTTTATATATATTATTTTGTCAATTAAATTCATATTTATTGAAAAACATTTTCAATAAATTTTTTTTTGTTATATGTACATTTT**

110 120 130 140 150 160 170 180 190 200

....|....|....|....|....|....|....|....|....|....|....|....|....|....|....|....|....|....|....|....|

**fus_quan_MIS** **-----------------------------------------------------------------------------------------------GCCCA**

**mar_MIS**  **-----------------GTCTTAAGGTTCATTTTGTTAAAATTAG-------------------------------------------------------**

**mar_quan_MIS** **----------------------------------------------------------------------------------------------------**

**Gfq_1**  **AAAATTATGTTGCGAATGTCTTAAGGTTCATTTTGTTAAAATTCATCATTATTGAGCGGAATTAAAACAGGAACCAAATTAACTTTTACCGTGCCGCCCA**

**Gfq_6**  **AAAATTATGTTGCGAATGTCTTAAGGTTCATTTTGTTAAAATTCATCATTATTGAGCGGAATTAAAACAGGAACCAAATTAACTTTTACCGTGCCGCCCA**

**Gfq_7**  **AAAATTATGTTGCGAAAGTCTTAAGGTTCATTTTGTTAAAATTCATCATTATTGAGCGGAATTAAAACAGGAACCAAATTAACTTTTACCGTGCCGCCCA**

**Gfq_Mad_1**  **AAAATTATGTTGCGAATGTCTTAAGGTTCATTTTGTTAAAATTCATCATTATTGAGCGGAATTAAAACAGGAACCAAATTAACTTTTACCGTGCCGCCCA**

**Gfq_Mad_2**  **AAAATTATGTTGCGAATGTCTTAAGGTTCATTTTGTTAAAATTCATCATTATTGAGCGGAATTAAAACAGGAACCAAATTAACTTTTACCGTGCCGCCCA**

**Gfq_Mad_3**  **AAAATTATGTTGCGAATGTCTTAAGGTTCATTTTGTTAAAATTCATCATTATTGAGCGGAATTAAAACAGGAACCAAATTAACTTTTACCGTGCCGCCCA**

**Gfq_Ben_2**  **AAAATTATGTTGCGAATGTCTTAAGGTTCATTTTGTTAAAATTCATCATTATTGAGCGGAATTAAAACAGGAACCAAATTAACTTTTACCGTGCCGCCCA**

**Gfq_Ben_3**  **AAAATTATGTTGCGAATGTCTTAAGGTTCATTTTGTTAAAATTCATCATTATTGAGCGGAATTAAAACAGGAACCAAATTAACTTTTACCGTGCCGCCCA**

**Gfq_Ben_4**  **AAAATTATGTTGCGAATGTCTTAAGGTTCATTTTGTTAAAATTCATCATTATTGAGCGGAATTAAAACAGGAACCAAATTAACTTTTACCGTGCCGCCCA**

**Gfq_Ben_5**  **AAAATTATGTTGCGAATGTCTTAAGGTTCATTTTGTTAAAATTCATCATTATTGAGCGGAATTAAAACAGGAACCAAATTAACTTTTACCGTGCCGCCCA**

**Gff_1**  **AAAATTATGTTGCGAATGTCTTAAGGTTCATTTTGTTAAAATTCATCATTATTGAGCGGAATTAAAACAGGAACCAAATTAACTTTTACCATGCCGCCCA**

**Gff_Buv_5**  **AAAATTATGTTGCGAATGTCTTAAGGTTCATTTTGTTAAAATTCATCATTATTGAGCGGAATTAAAACAGGAACCAAATTAACTTTTACCATGCCGCCCA**

**Gff_10**  **AAAATTATGTTGCGAATGTCTTAAGGTTCATTTTGTTAAAATTCATCATTATTGAGCGGAATTAAAACAGGAACCAAATTAACTTTTACCATGCCGCCCA**

**Gff_11**  **AAAATTATGTTGCGAATGTCTTAAGGTTCATTTTGTTAAAATTCATCATTATTGAGCGGAATTAAAACCGGAACCAAATTAACTTTTACCGTGCCGCCCA**

**Gff_12**  **AAAATTATGTTGCGAATGTCTTAAGGTTCATTTTGTTAAAATTCATCATTATTGAGCGGAATTAAAACAGGAACCAAATTAACTTTTACCATGCCGCCCA**

**Gff_7**  **AAAATTATGTTGCGAATGTCTTAAGGTTCATTTTGTTAAAATTCATCATTATTGAGCGGAATTAAAACAGGAACCAAATTAACTTTTACCRTGCCGCCCA**

**Gff_8**  **AAAATTATGTTGCGAATGTCTTAAGGTTCATTTTGTTAAAATTCATCATTATTGAGCGGAATTAAAACAGGAACCAAATTAACTTTTACCATGCCGCCCA**

**Gff_Gog_1**  **AAAATTATGTTGCGAATGTCTTAAGGTTCATTTTGTTAAAATTCATCATTATTGAGCGGAATTAAAACAGGAACCAAATTAACTTTTACCGTGCCGCCCA**

**Gff_Gog_2**  **AAAATTATGTTGCGAATGTCTTAAGGTTCATTTTGTTAAAATTCATCATTATTGAGCGGAATTAAAACAGGAACCAAATTAACTTTTACCGTGCCGCCCA**

**Gff_Kul_1**  **AAAATTATGTTGCGAATGTCTTAAGGTTCATTTTGTTAAAATTCATCATTATTGAGCGGAATTAAAACAGGAACCAAATTAACTTTTACCGTGCCGCCCA**

**Gff_Gog_9**  **AAAATTATGTTGCGAATGTCTTAAGGTTCATTTTGTTAAAATTCATCATTATTGAGCGGAATTAAAACAGGAACCAAATTAACTTTTACCGTGCCGCCCA**

**Gff_Moy_1**  **AAAATTATGTTGCGAATGTCTTAAGGTTCATTTTGTTAAAATTCATCATTATTGAGCGGAATTAAAACMGGAACCAAATTAACTTTTACCRTGCCGCCCA**

**Gff_Moy_2**  **AAAATTATGTTGCGAATGTCTTAAGGTTCATTTTGTTAAAATTCATCATTATTGAGCGGAATTAAAACMGGAACCAAATTAACTTTTACCGTGCCGCCCA**

**Gff_Moy_4**  **AAAATTATGTTGCGAATGTCTTAAGGTTCATTTTGTTAAAATTCATCATTATTGAGCGGAATTAAAACMGGAACCAAATTAACTTTTACCGTGCCGCCCA**

**Gfm_Kig_1**  **AAAATTATGTTGCGAATGTCTTAAGGTTCATTTTGTTAAAATTCGTCATTATTGAGCGGAATTAAAACAAGAACCAAATTAACTTTTACCGTGCCGCCCA**

**Gfm_Kig_3**  **AAAATTATGTTGCGAATGTCTTAAGGTTCATTTTGTTAAAATTCGTCATTATTGAGCGGAATTAAAACAAGAACCAAATTAACTTTTACCGTGCCGCCCA**

**Gfm_Kig_4**  **AAAATTATGTTGCGAATGTCTTAAGGTTCATTTTGTTAAAATTCGTCATTATTGAGCGGAATTAAAACAAGAACCAAATTAACTTTTACCGTGCCGCCCA**

**Gfm_Kig_6**  **AAAATTATGTTGCGAATGTCTTAAGGTTCATTTTGTTAAAATTCGTCATTATTGAGCGGAATTAAAACAAGAACCAAATTAACTTTTACCGTGCCGCCCA**

210 220 230 240 250 260 270 280 290 300

....|....|....|....|....|....|....|....|....|....|....|....|....|....|....|....|....|....|....|....|

**fus_quan_MIS** **TTTATTTTGGACTCC-------------------------------------------------------------------------------------**

**mar_MIS**  **----------------------------------------------------------------------------------------------------**

**mar_quan_MIS** **----------------------------------------------------------------------------------------------------**

**Gfq_1**  **TTTATTTTGGACTTCGGATTAAGTACAACATTTATTTTATATGTACAATATATATTATACGCATGGCGCATTTTTAGGCAAAAGGGTTAAAAAAAGCCTT**

**Gfq_6**  **TTTATTTTGGACTTCGGATTAAGTACAACATTTATTTTATATGTACAATATATATTATACGCATGGCGCATTTTTAGGCAAAAGGGTTAAAAAAAGCCTT**

**Gfq_7**  **TTTATTTTGGACTTCGGATTAAGTACAACATTTATTTTATATGTACAATATATATTATACGCATGGCGCATTTTTAGGCAAAAGGGTTAAAAAAAGCCTT**

**Gfq_Mad_1**  **TTTATTTTGGACTTCGGATTAAGTACAACATTTATTTTATATGTACAATATATATTATACGCATGGCGCATTTTTAGGCAAAAGGGTTAAAAAAAGCCTT**

**Gfq_Mad_2**  **TTTATTTTGGACTTCGGATTAAGTACAACATTTATTTTATATGTACAATATATATTATACGCATGGCGCATTTTTAGGCAAAAGGGTTAAAAAAAGCCTT**

**Gfq_Mad_3**  **TTTATTTTGGACTTCGGATTAAGTACAACATTTATTTTATATGTACAATATATATTATACGCATGGCGCATTTTTAGGCAAAAGGGTTAAAAAAAGCCTT**

**Gfq_Ben_2**  **TTTATTTTGGACTTCGGATTAAGTACAACATTTATTTTATATGTACAATATATATTATACGCATGGCGCATTTTTAGGCAAAAGGGTTAAAAAAAGCCTT**

**Gfq_Ben_3**  **TTTATTTTGGACTTCGGATTAAGTACAACATTTATTTTATATGTACAATATATATTATACGCATGGCGCATTTTTAGGCAAAAGGGTTAAAAAAAGCCTT**

**Gfq_Ben_4**  **TTTATTTTGGACTTCGGATTAAGTACAACATTTATTTTATATGTACAATATATATTATACGCATGGCGCATTTTTAGGCAAAAGGGTTAAAAAAAGCCTT**

**Gfq_Ben_5**  **TTTATTTTGGACTTCGGATTAAGTACAACATTTATTTTATATGTACAATATATATTATACGCATGGCGCATTTTTAGGCAAAAGGGTTAAAAAAAGCCTT**

**Gff_1**  **TTTATTTTGGACTTCGGATTAAGTACAACATTTATTTTATATGTACAATATATATTATACGCATGGCGCATTTTTAGGCAAAAGGGTTAAAAAAAGCCTT**

**Gff_Buv_5**  **TTTATTTTGGACTTCGGATTAAGTACAACATTTATTTTATATGTACAATATATATTATACGCATGGCGCATTTTTAGGCAAAAGGGTTAAAAAAAGCCTT**

**Gff_10**  **TTTATTTTGGACTTCGGATTAAGTACAACATTTATTTTATATGTACAATATATATTATACGCATGGCGCATTTTTAGGCAAAAGGGTTAAAAAAAGCCTT**

**Gff_11**  **TTTATTTTGGACTTCGGATTAAGTACAACATTTATTTTATATGTACAATATATATTATACGCATGGCGCATTTTTAGGCAAAAGGGTTAAAAAAAGCCTT**

**Gff_12**  **TTTATTTTGGACTTCGGATTAAGTACAACATTTATTTTATATGTACAATATATATTATACGCATGGCGCATTTTTAGGCAAAAGGGTTAAAAAAAGCCTT**

**Gff_7**  **TTTATTTTGGACTTCGGATTAAGTACAACATTTATTTTATATGTACAATATATATTATACGCATGGCGCATTTTTAGGCAAAAGGGTTAAAAAAAGCCTT**

**Gff_8**  **TTTATTTTGGACTTCGGATTAAGTACAACATTTATTTTATATGTACAATATATATTATACGCATGGCGCATTTTTAGGCAAAAGGGTTAAAAAAAGCCTT**

**Gff_Gog_1**  **TTTATTTTGGACTTCGGATTAAGTACAACATTTATTTTATATGTACAATATATATTATACGCATGGCGCATTTTTAGGCAAAAGGGTTAAAAAAAGCCTT**

**Gff_Gog_2**  **TTTATTTTGGACTTCGGATTAAGTACAACATTTATTTTATATGTACAATATATATTATACGCATGGCGCATTTTTAGGCAAAAGGGTTAAAAAAAGCCTT**

**Gff_Kul_1**  **TTTATTTTGGACTTCGGATTAAGTACAACATTTATTTTATATGTACAATATATATTATACGCATGGCGCATTTTTAGGCAAAAGGGTTAAAAAAAGCCTT**

**Gff_Gog_9**  **TTTATTTTGGACTTCGGATTAAGTACAACATTTATTTTATATGTACAATATATATTATACGCATGGCGCATTTTTAGGCAAAAGGGTTAAAAAAAGCCTT**

**Gff_Moy_1**  **TTTATTTTGGACTTCGGATTAAGTACAACATTTATTTTATATGTACAATATATATTATACGCATGGCGCATTTTTAGGCAAAAGGGTTAAAAAAAGCCTT**

**Gff_Moy_2**  **TTTATTTTGGACTTCGGATTAAGTACAACATTTATTTTATATGTACAATATATATTATACGCATGGCGCATTTTTAGGCAAAAGGGTTAAAAAAAGCCTT**

**Gff_Moy_4**  **TTTATTTTGGACTTCGGATTAAGTACAACATTTATTTTATATGTACAATATATATTATACGCATGGCGCATTTTTAGGCAAAAGGGTTAAAAAAAGCCTT**

**Gfm_Kig_1**  **TTTATTTTGGACTTAGGATTAAGTACAACATTTATTTTATATGTACAATATATATTATACGCATGGCGCATTTTTAGGCAAAAGGGTTAAAAAAAGCCTT**

**Gfm_Kig_3**  **TTTATTTTGGACTTAGGATTAAGTACAACATTTATTTTATATGTACAATATATATTATACGCATGGCGCATTTTTAGGCAAAAGGGTTAAAAAAAGCCTT**

**Gfm_Kig_4**  **TTTATTTTGGACTTAGGATTAAGTACAACATTTATTTTATATGTACAATATATATTATACGCATGGCGCATTTTTAGGCAAAAGGGTTAAAAAAAGCCTT**

**Gfm_Kig_6**  **TTTATTTTGGACTTAGGATTAAGTACAACATTTATTTTATATGTACAATATATATTATACGCATGGCGCATTTTTAGGCAAAAGGGTTAAAAAAAGCCTT**

310 320 330 340 350 360 370 380 390 400

....|....|....|....|....|....|....|....|....|....|....|....|....|....|....|....|....|....|....|....|

**fus_quan_MIS** **----------------------------------------------------------------------------------------------------**

**mar_MIS**  **----------------------------------------------------------------------------------------------------**

**mar_quan_MIS** **CCGTCAAAATCCCTTTTATATATTTAGG------------------------------------------------------------------------**

**Gfq_1**  **CCGTCAAAATCCCTTTTATATATTTATGTAATATTTAGCGTATTTTTAATTACGCATTTTTAGGCAAAAGGGTTAAAAAAA--GCCTTCCGTCAAATCCC**

**Gfq_6**  **CCGTCAAAATCCCTTTTATATATTTATGTAATATTTAGCGTATTTTTAATTACGCATTTTTAGGCAAAAGGGTTAAAAAAA--GCCTTCCGTCAAATCCC**

**Gfq_7**  **CCGTCAAAATCCCTTTTATATATTTATGTAATATTTAGCGTATTTTTAATTACGCATTTTTAGGCAAAAGGGTTAAAAAAA--GCCTTCCGTCAAATCCC**

**Gfq_Mad_1**  **CCGTCAAAATCCCTTTTATATATTTATGTAATATTTAGCGTATTTTTAATTACGCATTTTTAGGCAAAAGGGTTAAAAAAA--GCCTTCCGTCAAATCCC**

**Gfq_Mad_2**  **CCGTCAAAATCCCTTTTATATATTTATGTAATATTTAGCGTATTTTTAATTACGCATTTTTAGGCAAAAGGGTTAAAAAAA--GCCTTCCGTCAAATCCC**

**Gfq_Mad_3**  **CCGTCAAAATCCCTTTTATATATTTATGTAATATTTAGCGTATTTTTAATTACGCATTTTTAGGCAAAAGGGTTAAAAAAA--GCCTTCCGTCAAATCCC**

**Gfq_Ben_2**  **CCGTCAAAATCCCTTTTATATATTTATGTAATATTTAGCGTATTTTTAATTACGCATTTTTAGGCAAAAGGGTTAAAAAAA--GCCTTCCGTCAAATCCC**

**Gfq_Ben_3**  **CCGTCAAAATCCCTTTTATATATTTATGTAATATTTAGCGTATTTTTAATTACGCATTTTTAGGCAAAAGGGTTAAAAAAAAAGCCTTCCGTCAAATCCC**

**Gfq_Ben_4**  **CCGTCAAAATCCCTTTTATATATTTATGTAATATTTAGCGTATTTTTAATTACGCATTTTTAGGCAAAAGGGTTAAAAAAA--GCCTTCCGTCAAATCCC**

**Gfq_Ben_5**  **CCGTCAAAATCCCTTTTATATATTTATGTAATATTTAGCGTATTTTTAATTACGCATTTTTAGGCAAAAGGGTTAAAAAAA--GCCTTCCGTCAAATCCC**

**Gff_1**  **CCGTCAAAATCCCTTTTATATATTTATATAATATTTAGCGTATTTTTAATTACGCATTTTTAGGCAAAAGGGTTAAAAAAA--GCCTTCCGTCAAATCCC**

**Gff_Buv_5**  **CCGTCAAAATCCCTTTTATATATTTATATAATATTTAGCGTATTTTTAATTACGCATTTTTAGGCAAAAGGGTTAAAAAAA--GCCTTCCGTCAAATCCC**

**Gff_10**  **CCGTCAAAATCCCTTTTATATATTTATATAATATTTAGCGTATTTTTAATTACGCATTTTTAGGCAAAAGGGTTAAAAAAA--GCCTTCCGTCAAATCCC**

**Gff_11**  **CCGTCAAAATCCCTTTTATATATTTATATAATATTTAGCGTATTTTTAATTACGCATTTTTAGGCAAAAGGGTTAAAAAAA--GCCTTCCGTCAAATCCC**

**Gff_12**  **CCGTCAAAATCCCTTTTATATATTTATATAATATTTAGCGTATTTTTAATTACGCATTTTTAGGCAAAAGGGTTAAAAAAA--GCCTTCCGTCAAATCCC**

**Gff_7**  **CCGTCAAAATCCCTTTTATATATTTATATAATATTTAGCGTATTTTTAATTACGCATTTTTAGGCAAAAGGGTTAAAAAAA--GCCTTCCGTCAAATCCC**

**Gff_8**  **CCGTCAAAATCCCTTTTATATATTTATATAATATTTAGCGTATTTTTAATTACGCATTTTTAGGCAAAAGGGTTAAAAAAA--GCCTTCCGTCAAATCCC**

**Gff_Gog_1**  **CCGTCAAAATCCCTTTTATATATTTATATAATATTTAGCGTATTTTTAATTACGCATTTTTAGGCAAAAGGGTTAAAAAAA--GCCTTCCGTCAAATCCC**

**Gff_Gog_2**  **CCGTCAAAATCCCTTTTATATATTTATATAATATTTAGCGTATTTTTAATTACGCATTTTTAGGCAAAAGGGTTAAAAAAA--GCCTTCCGTCAAATCCC**

**Gff_Kul_1**  **CCGTCAAAATCCCTTTTATATATTTATATAATATTTAGCGTATTTTTAATTACGCATTTTTAGGCAAAAGGGTTAAAAAAA--GCCTTCCGTCAAATCCC**

**Gff_Gog_9**  **CCGTCAAAATCCCTTTTATATATTTATATAATATTTAGCGTATTTTTAATTACGCATTTTTAGGCAAAAGGGTTAAAAAAA--GCCTTCCGTCAAATCCC**

**Gff_Moy_1**  **CCGTCAAAATCCCTTTTATATATTTATATAATATTTAGCGTATTTTTAATTACGCATTTTTAGGCAAAAGGGTTAAAAAAA--GCCTTCCGTCAAATCCC**

**Gff_Moy_2**  **CCGTCAAAATCCCTTTTATATATTTATATAATATTTAGCGTATTTTTAATTACGCATTTTTAGGCAAAAGGGTTAAAAAAA--GCCTTCCGTCAAATCCC**

**Gff_Moy_4**  **CCGTCAAAATCCCTTTTATATATTTATATAATATTTAGCGTATTTTTAATTACGCATTTTTAGGCAAAAGGGTTAAAAAAA--GCCTTCCGTCAAATCCC**

**Gfm_Kig_1**  **CCGTCAAAATCCCTTTTATATATTTATGTAATATTTAGCGTATTTTTAATTACGCATTTTTAGGCAAAAGGGTTAAAAAAA--GCCTTCCGTCAAATCCC**

**Gfm_Kig_3**  **CCGTCAAAATCCCTTTTATATATTTATGTAATATTTAGCGTATTTTTAATTACGCATTTTTAGGCAAAAGGGTTAAAAAAA--GCCTTCCGTCAAATCCC**

**Gfm_Kig_4**  **CCGTCAAAATCCCTTTTATATATTTATGTAATATTTAGCGTATTTTTAATTACGCATTTTTAGGCAAAAGGGTTAAAAAAA--GCCTTCCGTCAAATCCC**

**Gfm_Kig_6**  **CCGTCAAAATCCCTTTTATATATTTATGTAATATTTAGCGTATTTTTAATTACGCATTTTTAGGCAAAAGGGTTAAAAAAA--GCCTTCCGTCAAATCCC**

410 420 430 440 450 460 470 480 490 500

....|....|....|....|....|....|....|....|....|....|....|....|....|....|....|....|....|....|....|....|

**fus_quan_MIS** **----------------------------------------------------------------------------------------------------**

**mar_MIS**  **----------------------------------------------------------------------------------------------------**

**mar_quan_MIS** **----------------------------------------------------------------------------------------------------**

**Gfq_1**  **TTTTTAAATTATGCACTTTCCAGCTTACCTTAATAGCGCATAATGATATTTGCATGTATTATTTGTGATTTGAAATTCATATCATATATGAATTGTTTCT**

**Gfq_6**  **TTTTTAAATTATGCACTTTCCAGCTTACCTTAATAGCGCATAATGATATTTGCATGTATTATTTGTGATTTGAAATTCATATCATATATGAATTGTTTCT**

**Gfq_7**  **TTTTTAAATTATGCACTTTCCAGCTTACCTTAATAGCGCATAATGATATTTGCATGTATTATTTGTGATTTGAAATTCATATCATATATGAATTATTTCT**

**Gfq_Mad_1**  **TTTTTAAATTATGCACTTTCCAGCTTACCTTAATAGCGCATAATGATATTTGCATGTATTATTTGTGATTTGAAATTCATATCATATATGAATTGTTTCT**

**Gfq_Mad_2**  **TTTTTAAATTATGCACTTTCCAGCTTACCTTAATAGCGCATAATGATATTTGCATGTATTATTTGTGATTTGAAATTCATATCATATATGAATTGTTTCT**

**Gfq_Mad_3**  **TTTTTAAATTATGCACTTTCCAGCTTACCTTAATAGCGCATAATGATATTTGCATGTATTATTTGTGATTTGAAATTCATATCATATATGAATTGTTTCT**

**Gfq_Ben_2**  **TTTTTAAATTATGCACTTTCCAGCTTACCTTAATAGCGCATAATGATATTTGCATGTATTATTTGTGATTTGAAATTCATATCATATATGAATTGTTTCT**

**Gfq_Ben_3**  **TTTTTAAATTATGCACTTTCCAGCTTACCTTAATAGCGCATAATGATATTTGCATGTATTATTTGTGATTTGAAATTCATATCATATATGAATTGTTTCT**

**Gfq_Ben_4**  **TTTTTAAATTATGCACTTTCCAGCTTACCTTAATAGCGCATAATGATATTTGCATGTATTATTTGTGATTTGAAATTCATATCATATATGAATTGTTTCT**

**Gfq_Ben_5**  **TTTTTAAATTATGCACTTTCCAGCTTACCTTAATAGCGCATAATGATATTTGCATGTATTATTTGTGATTTGAAATTCATATCATATATGAATTGTTTCT**

**Gff_1**  **TTTTTAAATTATGCACTTTCCAGCTTACCTTAATAGCGCATAATGATATTTGCATGTATTATTTGTGATTTGAAATTCATATCATATATGAATTGTTTCT**

**Gff_Buv_5**  **TTTTTAAATTATGCACTTTCCAGCTTACCTTAATAGCGCATAATGATATTTGCATGTATTATTTGTGATTTGAAATTCATATCATATATGAATTGTTTCT**

**Gff_10**  **TTTTTAAATTATGCACTTTCCAGCTTACCTTAATAGCGCATAATGATATTTGCATGTATTATTTGTGATTTGAAATTCATATCATATATGAATTGTTTCT**

**Gff_11**  **TTTTTAAATTATGCACTTTCCAGCTTACCTTAATAGCGCATAATGATATTTGCATGTATTATTTGTGATTTGAAATTCATATCATATATGAATTGTTTCT**

**Gff_12**  **TTTTTAAATTATGCACTTTCCAGCTTACCTTAATAGCGCATAATGATATTTGCATGTATTATTTGTGATTTGAAATTCATATCATATATGAATTGTTTCT**

**Gff_7**  **TTTTTAAATTATGCACTTTCCAGCTTACCTTAATAGCGCATAATGATATTTGCATGTATTATTTGTGATTTGAAATTCATATCATATATGAATTGTTTCT**

**Gff_8**  **TTTTTAAATTATGCACTTTCCAGCTTACCTTAATAGCGCATAATGATATTTGCATGTATTATTTGTGATTTGAAATTCATATCATATATGAATTGTTTCT**

**Gff_Gog_1**  **TTTTTAAATTATGCACTTTCCAGCTTACCTTAATAGCGCATAATGATATTTGCATGTATTATTTGTGATTTGAAATTCATATCATATATGAATTGTTTCT**

**Gff_Gog_2**  **TTTTTAAATTATGCACTTTCCAGCTTACCTTAATAGCGCATAATGATATTTGCATGTATTATTTGTGATTTGAAATTCATATCATATATGAATTGTTTCT**

**Gff_Kul_1**  **TTTTTAAATTATGCACTTTCCAGCTTACCTTAATAGCGCATAATGATATTTGCATGTATTATTTGTGATTTGAAATTCATATCATATATGAATTGTTTCT**

**Gff_Gog_9**  **TTTTTAAATTATGCACTTTCCAGCTTACCTTAATAGCGCATAATGATATTTGCATGTATTATTTGTGATTTGAAATTCATATCATATATGAATTGTTTCT**

**Gff_Moy_1**  **TTTTTAAATTATGCACTTTCCAGCTTACCTTAATAGCGCATAATGATATTTGCATGTATTATTTGTGATTTGAAATTCATATCATATATGAATTGTTTCT**

**Gff_Moy_2**  **TTTTTAAATTATGCACTTTCCAGCTTACCTTAATAGCGCATAATGATATTTGCATGTATTATTTGTGATTTGAAATTCATATCATATATGAATTGTTTCT**

**Gff_Moy_4**  **TTTTTAAATTATGCACTTTCCAGCTTACCTTAATAGCGCATAATGATATTTGCATGTATTATTTGTGATTTGAAATTCATATCATATATGAATTGTTTCT**

**Gfm_Kig_1**  **TTTTTAAATTATGCACTTTCCAGCTTACCTTAATAGCGCATAATGATATTTGCATGTATTATTTGTGATTTGAAATTCATATCATATATGAATTGTTTCT**

**Gfm_Kig_3**  **TTTTTAAATTATGCACTTTCCAGCTTACCTTAATAGCGCATAATGATATTTGCATGTATTATTTGTGATTTGAAATTCATATCATATATGAATTGTTTCT**

**Gfm_Kig_4**  **TTTTTAAATTATGCACTTTCCAGCTTACCTTAATAGCGCATAATGATATTTGCATGTATTATTTGTGATTTGAAATTCATATCATATATGAATTGTTTCT**

**Gfm_Kig_6**  **TTTTTAAATTATGCACTTTCCAGCTTACCTTAATAGCGCATAATGATATTTGCATGTATTATTTGTGATTTGAAATTCATATCATATATGAATTGTTTCT**

510 520 530 540

....|....|....|....|....|....|....|....|....|.

**fus_quan_MIS** **-----------------TTTAATGATCCTTCCGCAGG**

**mar_MIS**  **-----------------TTTAATGATCCTTCCGCAGG**

**mar_quan_MIS** **-----------------TTTAATGATCCTTCCGCAGG**

**Gfq_1**  **TTCGGATTGGAAACATCTTTAATGATCCTTCCGCAGGTTCACCTAC**

**Gfq_6**  **TTCGGATTGGAAACATCTTTAATGATCCTTCCGCAGGTTCACCTAC**

**Gfq_7**  **TTCGGATTGGAAACATCTTTAATGATCCTTCCGCAGGTTCACCTAC**

**Gfq_Mad_1**  **TTCGGATTGGAAACATCTTTAATGATCCTTCCGCAGGTTCACCTAC**

**Gfq_Mad_2**  **TTCGGATTGGAAACATCTTTAATGATCCTTCCGCAGGTTCACCTAC**

**Gfq_Mad_3**  **TTCGGATTGGAAACATCTTTAATGATCCTTCCGCAGGTTCACCTAC**

**Gfq_Ben_2**  **TTCGGATTGGAAACATCTTTAATGATCCTTCCGCAGGTTCACCTAC**

**Gfq_Ben_3**  **TTCGGATTGGAAACATCTTTAATGATCCTTCCGCAGGTTCACCTAC**

**Gfq_Ben_4**  **TTCGGATTGGAAACATCTTTAATGATCCTTCCGCAGGTTCACCTAC**

**Gfq_Ben_5**  **TTCGGATTGGAAACATCTTTAATGATCCTTCCGCAGGTTCACCTAC**

**Gff_1**  **TTCGGATTGGAAACATCTTTAATGATCCTTCCGCAGGTTCACCTAC**

**Gff_Buv_5**  **TTCGGATTGGAAACATCTTTAATGATCCTTCCGCAGGTTCACCTAC**

**Gff_10**  **TTCGGATTGGAAACATCTTTAATGATCCTTCCGCAGGTTCACCTAC**

**Gff_11**  **TTCGGATTGGAAACATCTTTAATGATCCTTCCGCAGGTTCACCTAC**

**Gff_12**  **TTCGGATTGGAAACATCTTTAATGATCCTTCCGCAGGTTCACCTAC**

**Gff_7**  **TTCGGATTGGAAACATCTTTAATGATCCTTCCGCAGGTTCACCTAC**

**Gff_8**  **TTCGGATTGGAAACATCTTTAATGATCCTTCCGCAGGTTCACCTAC**

**Gff_Gog_1**  **TTCGGATTGGAAACATCTTTAATGATCCTTCCGCAGGTTCACCTAC**

**Gff_Gog_2**  **TTCGGATTGGAAACATCTTTAATGATCCTTCCGCAGGTTCACCTAC**

**Gff_Kul_1**  **TTCGGATTGGAAACATCTTTAATGATCCTTCCGCAGGTTCACCTAC**

**Gff_Gog_9**  **TTCGGATTGGAAACATCTTTAATGATCCTTCCGCAGGTTCACCTAC**

**Gff_Moy_1**  **TTCGGATTGGAAACATCTTTAATGATCCTTCCGCAGGTTCACCTAC**

**Gff_Moy_2**  **TTCGGATTGGAAACATCTTTAATGATCCTTCCGCAGGTTCACCTAC**

**Gff_Moy_4**  **TTCGGATTGGAAACATCTTTAATGATCCTTCCGCAGGTTCACCTAC**

**Gfm_Kig_1**  **TTCGGATTGGAAACATCTTTAATGATCCTTCCGCAGGTTCACCTAC**

**Gfm_Kig_3**  **TTCGGATTGGAAACATCTTTAATGATCCTTCCGCAGGTTCACCTAC**

**Gfm_Kig_4**  **TTCGGATTGGAAACATCTTTAATGATCCTTCCGCAGGTTCACCTAC**

**Gfm_Kig_6**  **TTCGGATTGGAAACATCTTTAATGATCCTTCCGCAGGTTCACCTAC**
