# Supplementary figures and images for: Cryptic Diversity within the Major Trypanosomiasis Vector Glossina fuscipes Revealed by Molecular Markers
Source: PLoS Negl Trop Dis. 2011 Aug 9;5(8):e1266. doi: 10.1371/journal.pntd.0001266 (PMC3153427; doi:10.1371/journal.pntd.0001266)

A

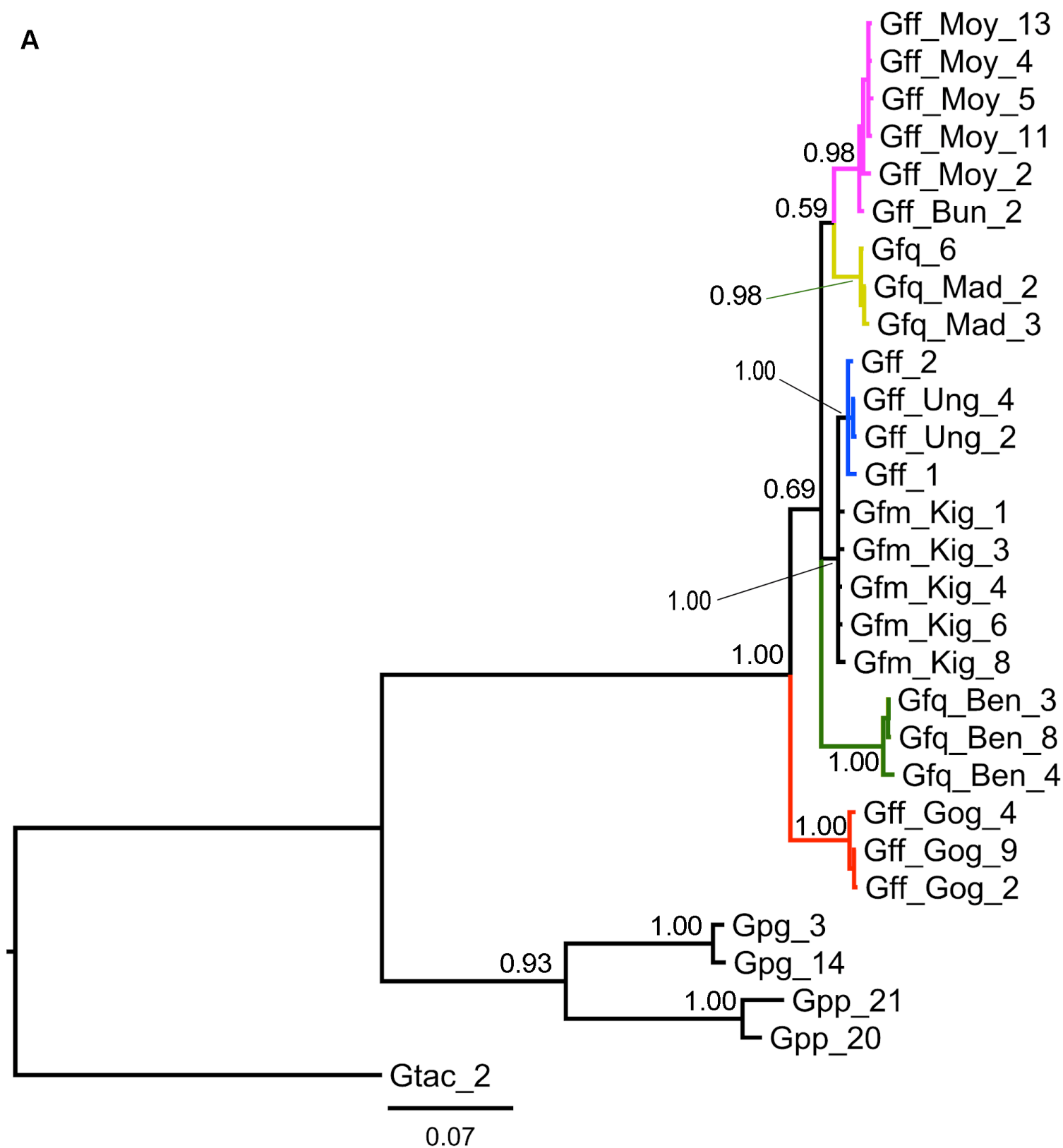

B

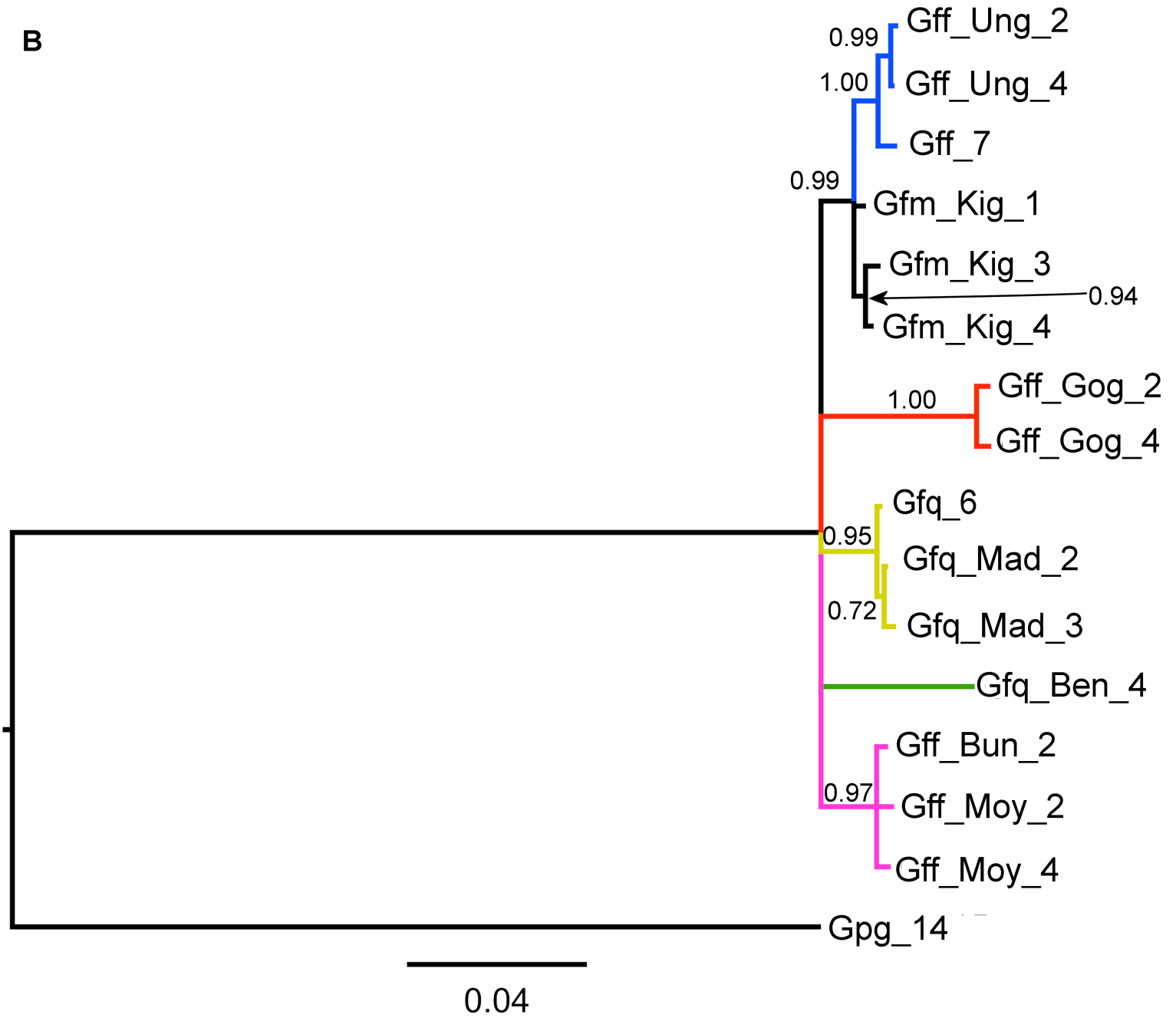

C

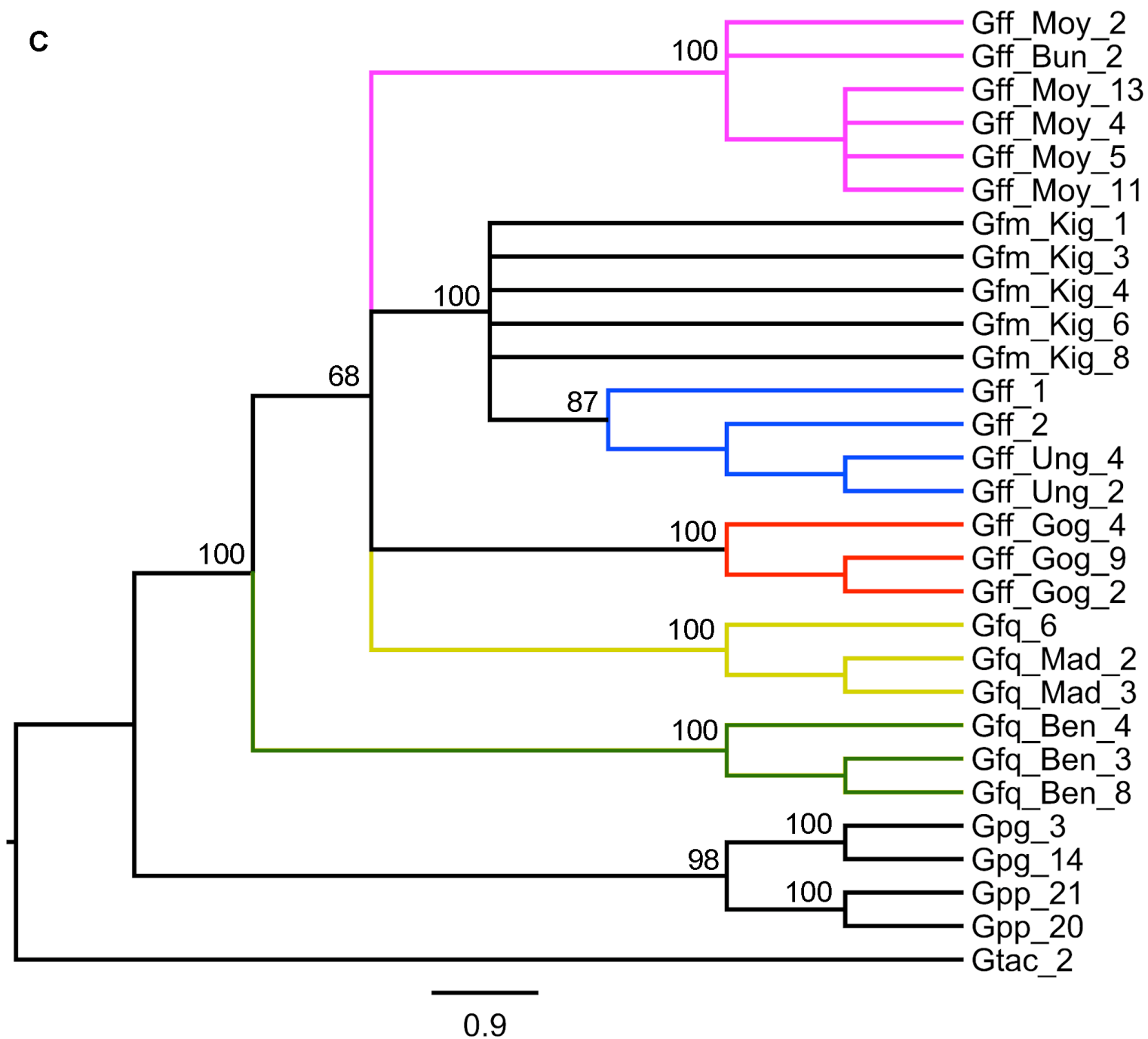

Supplement: Figure S3 — Bayesian and distance based neighbour-joining phylogenies based upon sequence data from the mtDNA COI+ND2 genes. Branch colour reflects sample collection location blue: Lake Victoria Basin; black; G.f.martinii from Tanzania; red: Ethiopia; pink: Mid/Northern Uganda; yellow: west DRC, green, Bena Tschibangu. A. Bayesian 29 taxa (full data set) used for Bayesian Phylogeny testing. Branch support is given as posterior probability. B. Bayesian phylogeny of the 16 taxa used for Bayesian Phylogeny testing. Branch support is given as posterior probability. C. Neighbour joining tree, based on Tamura and Nei (Tamura and Nei 1993. Molecular Biology and Evolution 10, pp 512–526) corrected distances. Branch support is shown as a percentage of 2000 bootstrap replicates. (PDF) [file pntd.0001266.s003.pdf]

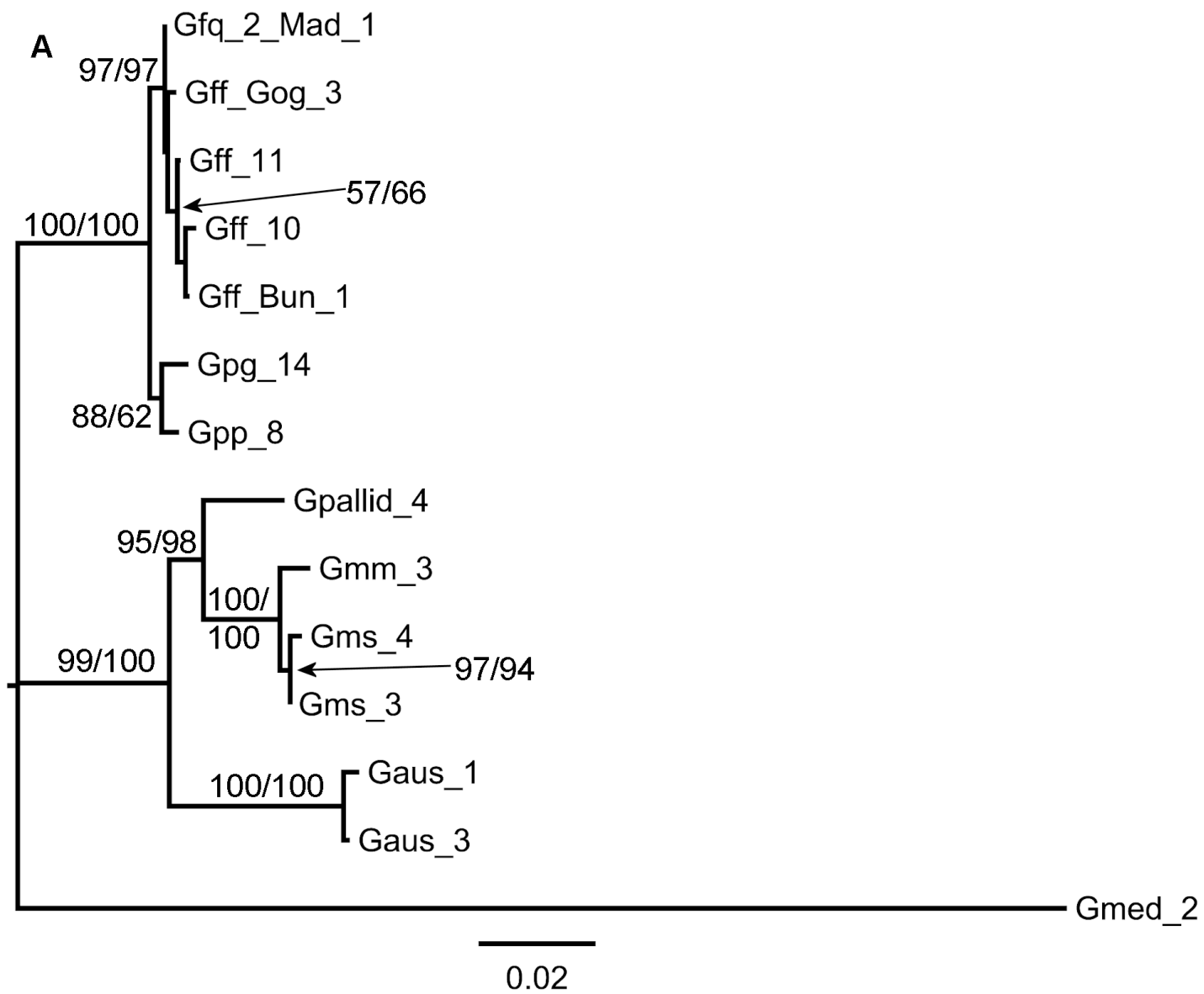

B

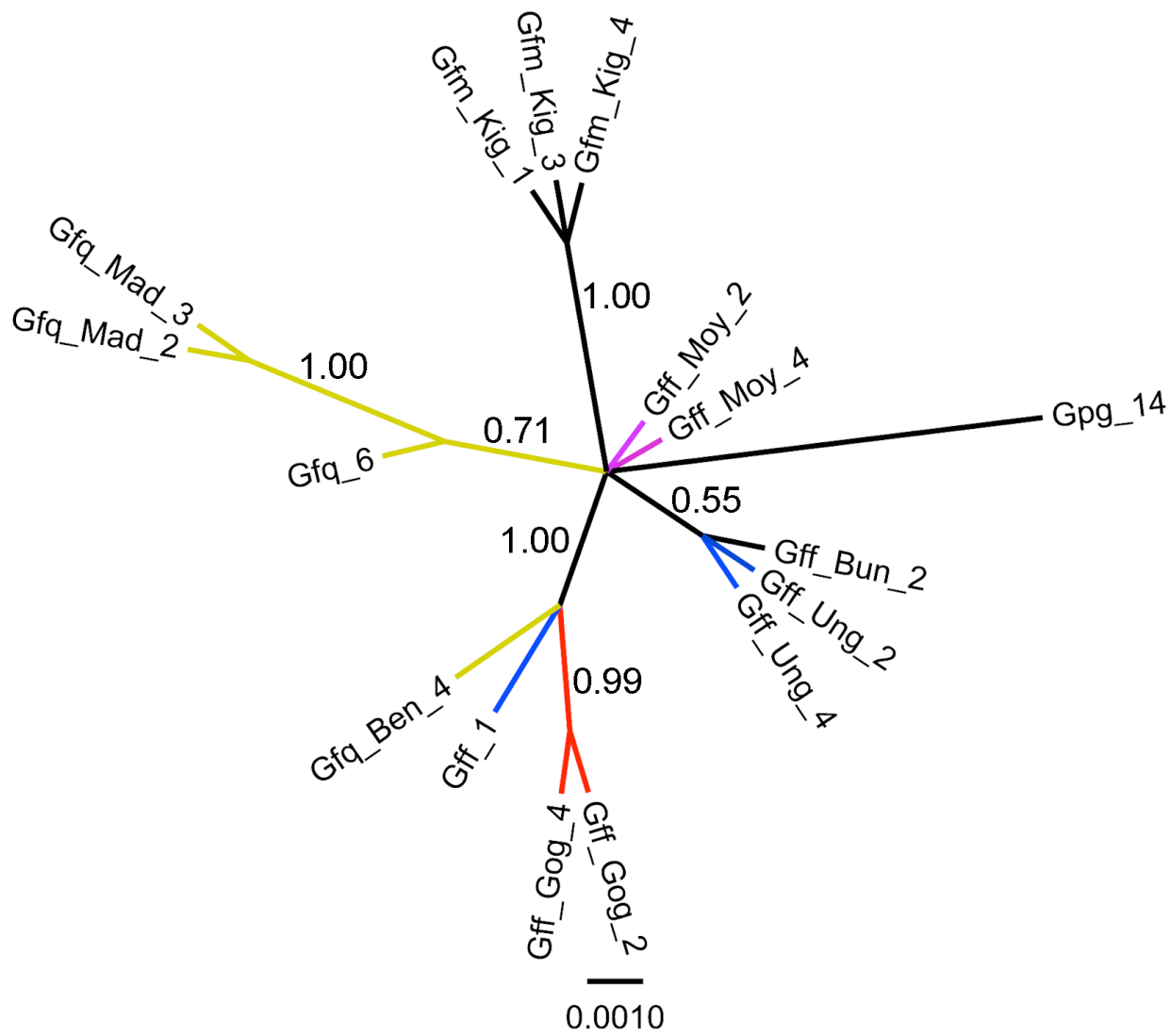

Supplement: Figure S4 — Bayesian phylogeny based upon sequence data from the Wigglesworthia locus, YcfW (14 taxa data set). Used for Bayesian and Shimodaira-Hasegawa tests (Shimodaira and Hasegawa 1999. Molecular Biology and Evolution 16, pp 1114–1116). Branch support is given as posterior probability. See Table S1 for key to specimen names. (PDF) [file pntd.0001266.s004.pdf]

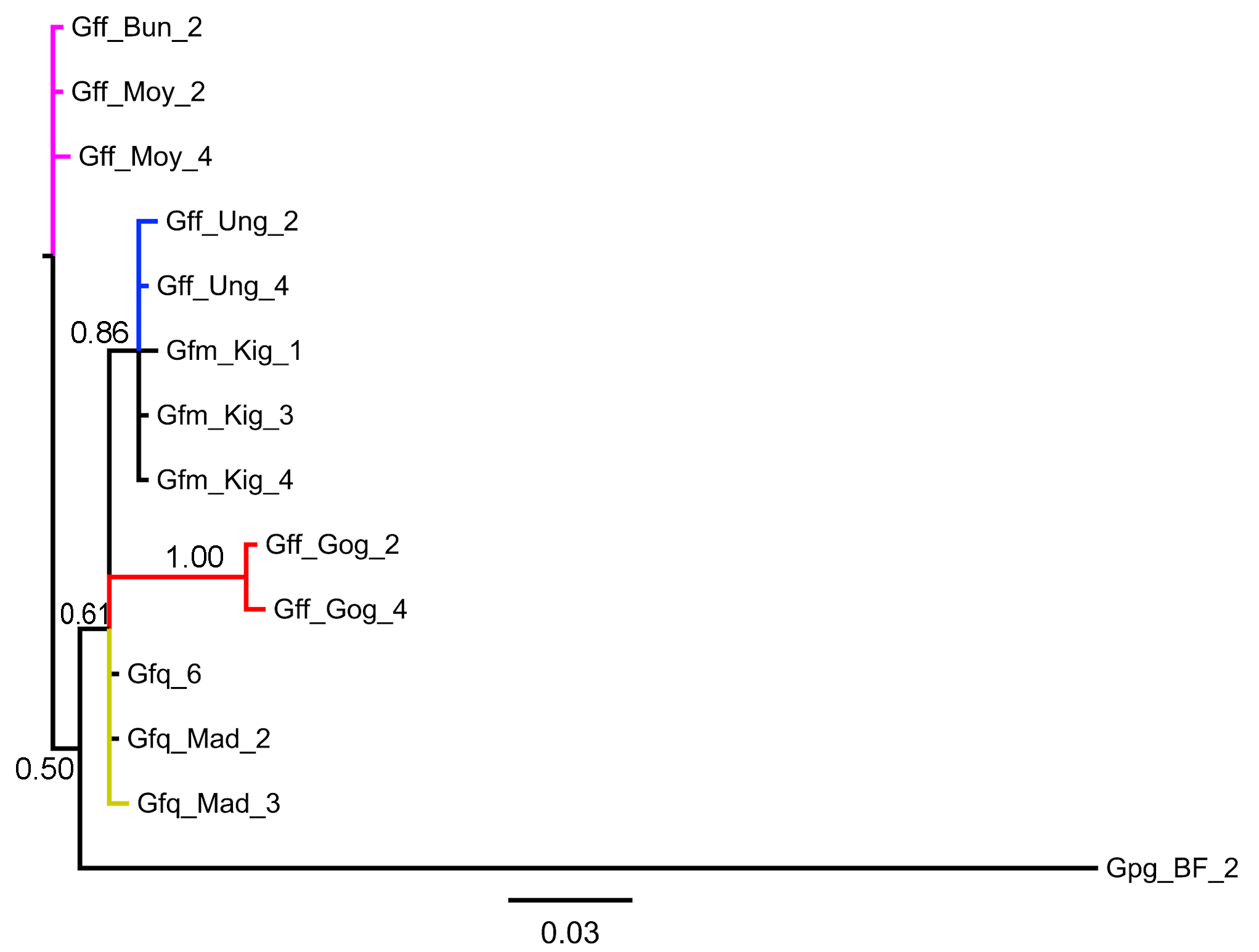

Supplement: Figure S5 — Bayesian, maximum likelihood and distance based neighbour-joining phylogenies based upon sequence data from the nDNA Period gene. A. Gene tree for 2070 bp of period gene from selected taxa from genus Glossina. Node support for maximum likelihood and distance neighbour joining trees are given as a percentage of 1000 and 2000 bootstrap replicates respectively. B. Bayesian phylogeny for 5′ end of period gene (880 bp alignment) used for Bayesian and Shimodaira-Hasegawa tests (Shimodaira and Hasegawa 1999. Molecular Biology and Evolution 16, pp 1114–1116). Branch support is given as posterior probability. See Table S1 for key to specimen names. (TIF) [file pntd.0001266.s005.tif]

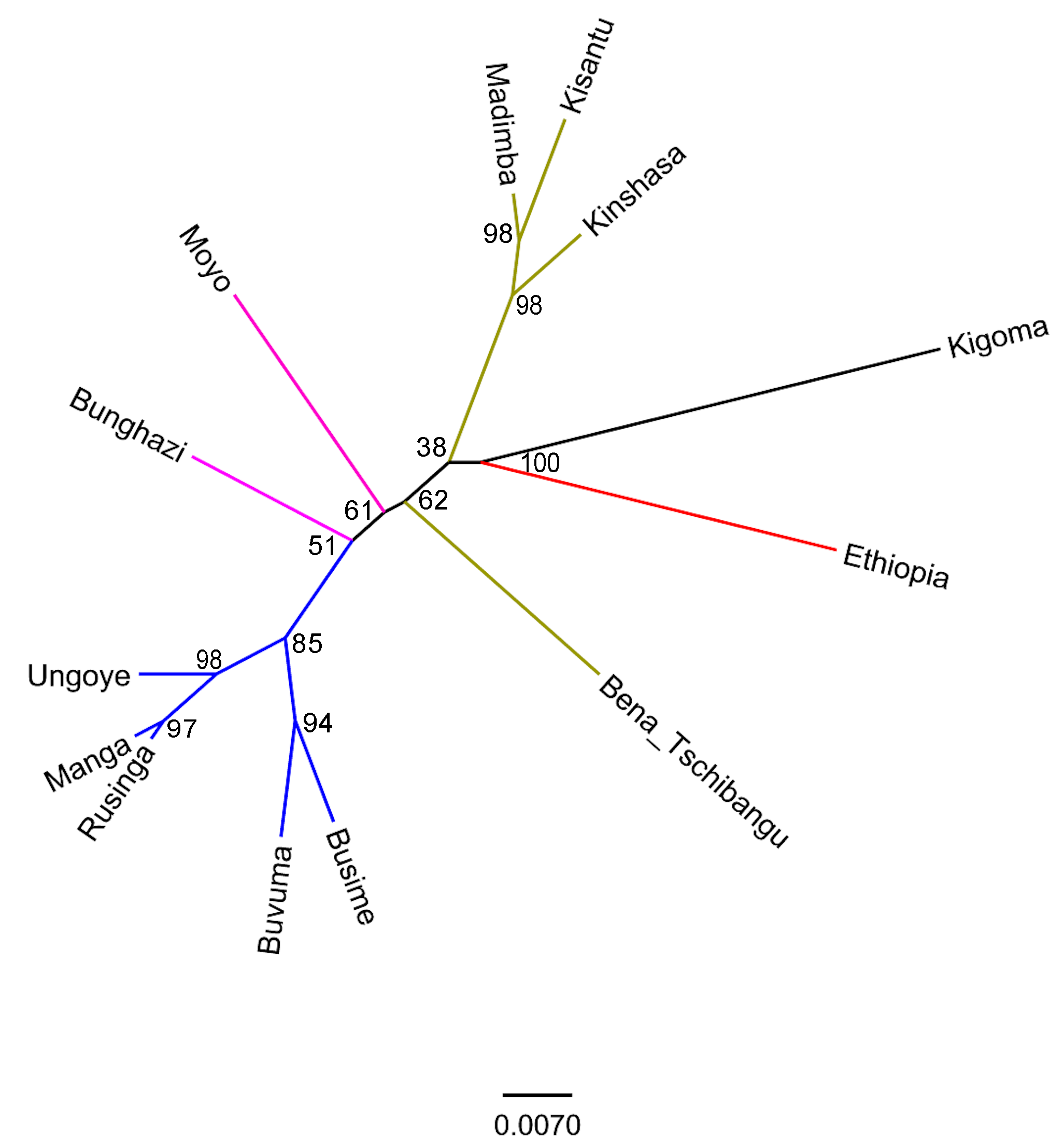

Supplement: Figure S6 — Neighbour-joining tree using Cavalli-Svorza distances for the microsatellite data set. Cavalli-Svorza distances were calculated from ENA (Excluding Null Alleles: Chapuis and Estoup 2007. Molecular Biology and Evolution 24, pp 621–631) corrected genotype data. Node support values are the proportion of 1000 bootstrap replicates over loci supporting that node. Branch colour reflects sample collection location blue: Lake Victoria Basin; black; G. f. martinii from Tanzania; red: Ethiopia; pink: Mid/Northern Uganda; green: DRC. (TIF) [file pntd.0001266.s006.tif]
