# Supplementary material for: Cryptic Diversity within the Major Trypanosomiasis Vector Glossina fuscipes Revealed by Molecular Markers
Source: PLoS Negl Trop Dis. 2011 Aug 9;5(8):e1266. doi: 10.1371/journal.pntd.0001266 (PMC3153427; doi:10.1371/journal.pntd.0001266)
Supplement: Table S1 — Accession numbers for all sequence data. (DOC) [file pntd.0001266.s007.doc]

### Table S1. Accession numbers for all sequence data

| **Collection location** | **NCBI name** | ***COI*** | ***ND2*** | ***YcfW*** | ***Period*** | ***ITS1*** |
| --- | --- | --- | --- | --- | --- | --- |
| DRC, Kinshasa | Gfq_1 | HQ387026 |  | HQ387078 |  | EU591941 |
| DRC, Kinshasa | Gfq_2 |  |  |  |  |  |
| DRC, Kinshasa | Gfq_18 | HQ387027 |  |  |  |  |
| DRC, Kinshasa | Gfq_3 | same as EU591871 |  | same as HQ387077 |  |  |
| DRC, Kinshasa | Gfq_5 | HQ387028 | EU591893 |  |  |  |
| DRC, Kinshasa | Gfq_6 | EU591870 | same as EU591893 | same as HQ387077 | HQ387101 | same as EU591941 |
| DRC, Kinshasa | Gfq_7 | same as EU591870 | same as EU591893 |  |  | EU591942 |
| DRC, Kinshasa | Gfq_8 | same as EU591870 |  |  |  |  |
| DRC, Kinshasa | Gfq_9 | same as EU591870 |  |  |  |  |
| DRC, Kinshasa | Gfq_10 | same as EU591870 |  |  |  |  |
| DRC, Kinshasa | Gfq_11 | same as EU591870 |  |  |  |  |
| DRC, Kinshasa | Gfq_12 | same as EU591870 |  |  |  |  |
| DRC, Kinshasa | Gfq_13 | EU591871 |  |  |  |  |
| DRC, Kinshasa | Gfq_14 | HQ387029 |  |  |  |  |
| DRC, Kinshasa | Gfq_19 | HQ387030 |  | same as HQ387077 |  |  |
| DRC, Madimba | Gfq_Mad_1 | HQ387031 |  | HQ387076 | HQ387125* | same as EU591941 |
| DRC, Madimba | Gfq_Mad_2 | HQ387032 | same as EU591893 | HQ387077 | HQ387103 | same as EU591941 |
| DRC, Madimba | Gfq_Mad_3 | HQ387033 | HQ387085 | same as HQ387077 | same as HQ387103 | same as EU591941 |
| DRC, Madimba | Gfq_Mad_4 | HQ387034 |  | same as HQ387076 |  |  |
| DRC, Madimba | Gfq_Mad_5 | same as HQ387033 |  | same as HQ387077 |  |  |
| DRC, Madimba | Gfq_Mad_6 | HQ387035 |  | same as HQ387077 |  |  |
| DRC, Madimba | Gfq_Mad_7 | same as HQ387032 |  | same as HQ387078 |  |  |
| DRC, Madimba | Gfq_Mad_8 | same as HQ387033 |  |  |  |  |
| DRC, Madimba | Gfq_Mad_9 | same as HQ387032 |  |  |  |  |
| DRC, Madimba | Gfq_Mad_10 | same as HQ387032 |  |  |  |  |
| DRC, Bena Tschibangu | Gfq_Ben_1 | HQ387036 |  |  |  |  |
| DRC, Bena Tschibangu | Gfq_Ben_2 | HQ387037 |  |  | HQ387104 | same as EU591941 |
| DRC, Bena Tschibangu | Gfq_Ben_3 | same as HQ387036 | HQ387086 |  |  | same as EU591941 |
| DRC, Bena Tschibangu | Gfq_Ben_4 | HQ387038 | HQ387087 |  | HQ387105 | same as EU591941 |
| DRC, Bena Tschibangu | Gfq_Ben_5 | same as HQ387036 |  |  |  | same as EU591941 |
| DRC, Bena Tschibangu | Gfq_Ben_6 | same as HQ387038 |  |  |  |  |
| DRC, Bena Tschibangu | Gfq_Ben_7 | same as HQ387036 |  |  |  |  |
| DRC, Bena Tschibangu | Gfq_Ben_8 | HQ387039 | same as HQ387086 |  |  |  |
| DRC, Bena Tschibangu | Gfq_Ben_9 | HQ387040 |  |  |  |  |
| DRC, Bena Tschibangu | Gfq_Ben_10 | same as HQ387036 |  |  |  |  |
| Ethiopia, Gogara | Gff_Gog_1 | HQ387041 |  | HQ387081 | HQ387106 | HQ387129 |
| Ethiopia, Gogara | Gff_Gog_2 | HQ387042 | HQ387088 | HQ387082 | same as HQ387106 | same as HQ387129 |
| Ethiopia, Gogara | Gff_Gog_3 | same as HQ387042 |  |  | HQ387128 |  |
| Ethiopia, Gogara | Gff_Gog_5 | same as HQ387042 |  |  |  |  |
| Ethiopia, Gogara | Gff_Gog_6 | HQ387043 |  | same as HQ387082 |  |  |
| Ethiopia, Gogara | Gff_Gog_7 | HQ387044 |  | HQ387084 |  |  |
| Ethiopia, Gogara | Gff_Gog_8 | HQ387045 |  | same as HQ387081 |  |  |
| Ethiopia, Gogara | Gff_Gog_9 | same as HQ387042 | HQ387089 |  |  | same as HQ387129 |
| Ethiopia, Deme | Gff_Dem_1 | HQ387046 |  | same as HQ387082 |  |  |
| Ethiopia, Kulano | Gff_Kul_1 | same as HQ387041 | HQ387090 | HQ387083 | same as HQ387106 | same as HQ387129 |
| Uganda, Moyo | Gff_Moy_1 | HQ387047 |  | same as HQ387064 | HQ387107 (Hz) | HQ387130 |
| Uganda, Moyo | Gff_Moy_2 | same as HQ387051 | HQ387091 | same as HQ387064 | same as HQ387107 | HQ387131 |
| Uganda, Moyo | Gff_Moy_3 | same as HQ387051 | same as HQ387091 | same as HQ387064 |  |  |
| Uganda, Moyo | Gff_Moy_4 | HQ387048 | HQ387092 | HQ387065 | same as HQ387107 | same as HQ387131 |
| Uganda, Moyo | Gff_Moy_5 | HQ387049 | HQ387093 | HQ387066 |  |  |
| Uganda, Moyo | Gff_Moy_6 | same as HQ387047 |  | same as HQ387064 |  |  |
| Uganda, Moyo | Gff_Moy_7 |  |  | same as HQ387064 |  |  |
| Uganda, Moyo | Gff_Moy_8 |  |  | same as HQ387064 |  |  |
| Uganda, Moyo | Gff_Moy_9 | same as HQ387048 | same as HQ387092 | same as HQ387065 |  |  |
| Uganda, Moyo | Gff_Moy_10 | same as HQ387048 | same as HQ387092 |  |  |  |
| Uganda, Moyo | Gff_Moy_11 | same as HQ387047 | same as HQ387092 | same as HQ387064 |  |  |
| Uganda, Moyo | Gff_Moy_12 | same as HQ387048 | same as HQ387092 | same as HQ387065 |  |  |
| Uganda, Moyo | Gff_Moy_13 | HQ387050 | same as HQ387092 | same as HQ387066 |  |  |
| Uganda, Bunghazi | Gff_Bun_1 |  |  | HQ387124 | HQ387124 |  |
| Uganda, Bunghazi | Gff_Bun_2 | same as EU591828 | same as HQ387094 | HQ387064 | HQ387108 |  |
| Uganda, Bunghazi | Gff_Bun_3 | HQ387051 |  |  |  |  |
| Uganda, Bunghazi | Gff_Bun_4 | same as EU591828 |  |  |  |  |
| Uganda, Bunghazi | Gff_Bun_5 | same as EU591828 |  | same as HQ387066 |  |  |
| Uganda, Bunghazi | Gff_Bun_6 |  |  | same as HQ387068 |  |  |
| Uganda, Bunghazi | Gff_Bun_7 | same as HQ387051 |  |  |  |  |
| Uganda, Bunghazi | Gff_Bun_8 | same as EU591828 |  |  | same as HQ387110 |  |
| Uganda, Bunghazi | Gff_Bun_9 | HQ387052 |  |  |  |  |
| Uganda, Bunghazi | Gff_Bun_10 | HQ387053 |  |  |  |  |
| Uganda, Bunghazi | Gff_10 | EU591828 | same as HQ387094 |  | HQ387127 | HQ387132 |
| Uganda, Bunghazi | Gff_11 | same as EU591828 | HQ387094 |  | HQ387126 | EU591939 |
| Uganda, Buvuma Is. | Gff_1 | EU591876 | EU591901 |  | HQ387109 | same as HQ387132 |
| Uganda, Buvuma Is. | Gff_Buv_5 |  |  |  |  | same as HQ387132 |
| Uganda, Buvuma Is. | Gff_2 | EU591877 | EU591902 | same as HQ387068 |  |  |
| Uganda, Buvuma Is. | Gff_Buv_1 | same as 21258 | EU591903 |  |  |  |
| Uganda, Buvuma Is. | Gff_Buv_2 | same as EU591877 |  |  |  |  |
| Uganda, Buvuma Is. | Gff_Buv_3 | same as HQ387055 |  |  |  |  |
| Uganda, Buvuma Is. | Gff_Buv_4 | same as HQ387055 |  |  |  |  |
| Kenya, Ungoye | Gff_Ung_1 | same as HQ387055 |  |  |  |  |
| Kenya, Ungoye | Gff_Ung_2 | same as HQ387054 | same as HQ387095 |  | HQ387110 |  |
| Kenya, Ungoye | Gff_Ung_3 | same as HQ387054 |  |  |  |  |
| Kenya, Ungoye | Gff_Ung_4 | same as HQ387055 | HQ387095 |  | same as HQ387110 |  |
| Kenya, Ungoye | Gff_Ung_5 | same as HQ387054 |  |  |  |  |
| Kenya, Ungoye | Gff_Ung_6 | same as HQ387054 |  |  |  |  |
| Kenya, Ungoye | Gff_Ung_7 | same as HQ387054 |  |  |  |  |
| Kenya, Ungoye | Gff_Ung_8 | same as HQ387055 |  |  |  |  |
| Kenya, Ungoye | Gff_Ung_9 | HQ387054 |  | HQ387079 |  |  |
| Kenya, Ungoye | Gff_Ung_10 | HQ387055 |  | same as HQ387068 |  |  |
| Kenya, Ungoye | Gff_Ung_11 | same as HQ387054 |  |  |  |  |
| Kenya, Ungoye | Gff_Ung_12 | same as HQ387054 |  |  |  |  |
| Kenya, Ungoye | Gff_Ung_13 | same as HQ387055 |  | HQ387080 |  |  |
| Kenya, Ungoye | Gff_Ung_14 | same as HQ387054 |  |  |  |  |
| Kenya, Ungoye | Gff_Ung_15 | HQ387056 |  |  |  |  |
| Kenya, Manga Is | Gff_7 | EU591826 | EU_591899 |  |  | same as HQ387130 |
| Kenya, Manga Is | Gff_8 |  | EU_591900 |  |  | same as HQ387132 |
| Kenya, Manga Is | Gff_Man_1 | same as HQ387055 | same as EU_591900 |  |  |  |
| Kenya, Chamaunga Is | Gff_Cha_1 | same as HQ387055 |  |  |  |  |
| Kenya, Chamaunga Is | Gff_Cha_2 |  | same as EU_591900 |  |  |  |
| Kenya, Chamaunga Is | Gff_Cha_3 |  | same as EU_591900 |  |  |  |
| Tanzania,  Kigoma | Gfm_Kig_1 | HQ387057 | HQ387096 | HQ387067 | HQ387111 | HQ387133 |
| Tanzania,  Kigoma | Gfm_Kig_2 | same as HQ387057 |  |  |  |  |
| Tanzania,  Kigoma | Gfm_Kig_3 | HQ387058 | HQ387097 | HQ387068 | HQ387112 | same as HQ387133 |
| Tanzania,  Kigoma | Gfm_Kig_4 | HQ387059 | HQ387098 | same as HQ387068 | same as HQ387112 | same as HQ387133 |
| Tanzania,  Kigoma | Gfm_Kig_5 | HQ387060 |  |  |  |  |
| Tanzania,  Kigoma | Gfm_Kig_6 | HQ387061 | HQ387099 | same as HQ387068 |  | same as HQ387133 |
| Tanzania,  Kigoma | Gfm_Kig_7 | same as HQ387059 |  |  |  |  |
| Tanzania,  Kigoma | Gfm_Kig_8 | HQ387062 | HQ387100 | HQ387069 |  |  |
| Tanzania,  Kigoma | Gfm_Kig_9 | same as HQ387059 |  |  |  |  |
| Tanzania,  Kigoma | Gfm_Kig_10 | HQ387063 |  |  |  |  |
| Uganda, Bukhubalo | Gff_12 |  |  |  |  | same as HQ387132 |
| B. Faso,  *G.p.gambiensis* | Gpg_BF_1 |  |  | HQ387071 |  |  |
| B. Faso,  *G.p.gambiensis* | Gpg_14 | EU591855 | EU591887 |  | HQ387121 |  |
| B. Faso,  *G.p.gambiensis* | Gpg_BF_2 |  |  | HQ387070 |  |  |
| Guinea,  *G.p.gambiensis* | Gpg_Gui_1 |  |  | HQ387072 |  |  |
| Cote d’ Ivoire,  *G.p.palpalis* | Gpp_8 |  |  |  | HQ387120 |  |
| Cote d’ Ivoire,  *G.p.palpalis* | Gpp_29 |  |  | HQ387075 |  |  |
| Cameroon,  *G.p.palpalis* | Gpp_20 | EU591865 | EU591898 |  |  |  |
| Colony,  *G.p.palpalis* | Gpp_col_1 |  |  | HQ387073 |  |  |
| Eq. Guinea,  *G.p.palpalis* | Gpp_21 | EU591825 | EU591905 |  |  |  |
| Cameroon,  *G.p.palpalis* | Gpp_27 |  |  | HQ387074 |  |  |
| Colony,  *G. austeni* | Gaus_1 |  |  |  | HQ387115 |  |
| Colony,  *G. austeni* | Gaus_3 |  |  |  | HQ387116 |  |
| Burkina Faso  *G.medicorum* | Gmed_2 |  |  |  | HQ387117 |  |
| Colony,  *G.m.submorsitans* | Gms_3 |  |  |  | HQ387118 |  |
| Colony,  *G.m.submorsitans* | Gms_4 |  |  |  | HQ387119 |  |
| Colony LSTM  *G.m.morsitans* | Gmm_3 |  |  |  | HQ387122 |  |
| Zimbabwe  *G.pallidipes* | Gpallid_4 |  |  |  | HQ387123 |  |

**Footnote**. Grey shading indicates that the sequence has already been published and was either reanalysed or found in a new individual in this study. For sequences labelled "same as", this indicates that it is identical to the individual with the same accession number submitted to Genbank. Hz=heterozygous. There were three collections made on adjacent rivers in Ethiopia (Gogara, Deme and Kulano), this collection information is retained in the Genbank accession number but for simplicity in microsatellite analyses all Ethiopian specimens are referred to as Gogara River (Gog). For the *Period* gene the submitted sequences differ in length between specimens because additional sequence information was obtained for some specimens during exploratory work to determine the most appropriate section of the gene for phylogenetic studies.

*HQ387128 is a composite sequence derived frm Gfq_2 and Gfq_Mad_1
