# Supplementary material for: Cryptic Diversity within the Major Trypanosomiasis Vector Glossina fuscipes Revealed by Molecular Markers
Source: PLoS Negl Trop Dis. 2011 Aug 9;5(8):e1266. doi: 10.1371/journal.pntd.0001266 (PMC3153427; doi:10.1371/journal.pntd.0001266)
Supplement: Table S2 — PCR conditions for amplification of the Glossina Period gene (A) and microsatellites (B). (DOC) [file pntd.0001266.s008.doc]

Table S2: PCR conditions for amplification of the *Glossina* *Period* gene (A) and microsatellites (B).

(A)

| **Forward primer** | **Reverse primer** | **[MgCl_2_], mM** | **Temperature cycles** |
| --- | --- | --- | --- |
| GlossPerfor2 | GlossPerrev2 | 3 | 94°C for 30 seconds  54.8°C for 30 seconds  72°C for 2 minutes |
| GlossPerfor3 | GlossPerrev3 | 3 | 94°C for 30 seconds  53°C for 30 seconds  72°C for 2 minutes |
| Permiddegenfor2 | Permiddegenrev2 | 1.5 | 95°C for 30 seconds  57°C for 30 seconds  72°C for 90 seconds |

(B)

| **Locus** | **Primer sequences** | **Allele size range and repeat motif** | **Label** |
| --- | --- | --- | --- |
| GfA3 | **M13GfA3F**: 5’-**CACGACGTTGTAAAACGAC**AGCCGCTTTAAGTTTGTTGC-3’ | 144-276 (CA) | IR700 |
|  | **GfA3R**: 5’-AACTCGTTTGTTCGGTGGGC-3’ |  |  |
| GfB8 | **M13GfB8F**: 5’-**CACGACGTTGTAAAACGAC**TGCTACCTCCCTGCTATCTC-3’ | 146-218 (CT) | IR800 |
|  | **GfB8R**: 5’-TCACTTTCCCGAGCTTAGAG-3’ |  |  |
| GfB101 | **M13GfB101F**: 5’-**CACGACGTTGTAAAACGAC**GACTTTTTAGAGCCAGTGCC-3’ | 108-187 (GA) | IR700 |
|  | **GfB101R**: 5’-CTTAATCTTTAGGAAGTCAC-3’ |  |  |
| GfB105 | **M13GfB105F**: 5’-**CACGACGTTGTAAAACGAC**GCCGATATTTGTTGAAAGCAT-3’ | 120-268 (CT) | IR700 |
|  | **GfB105R**: 5’-CCCTTAACGAATCACATTTGC-3’ |  |  |
| A10 | **M13A10F**: 5-’**CACGACGTTGTAAAACGAC**GCAACGCCAAGTGAAATAAAG-3’ | 190-226 (CA) | IR800 |
|  | **A10R**: 5’-TACTGGGCTCGCGTACATAAT-3’ |  |  |
