# Supplementary material for: Cryptic Diversity within the Major Trypanosomiasis Vector Glossina fuscipes Revealed by Molecular Markers
Source: PLoS Negl Trop Dis. 2011 Aug 9;5(8):e1266. doi: 10.1371/journal.pntd.0001266 (PMC3153427; doi:10.1371/journal.pntd.0001266)
Supplement: Table S3 — Results of Shimodaira-Hasegawa tests of monophyly. (DOC) [file pntd.0001266.s009.doc]

Table S3: Results of Shimodaira-Hasegawa tests of monophyly.

| Group | COI+ND2 (mtDNA) | | | | | | YcfW (Wigglesworthia) | | | Period | | | ITS1 | | |
| --- | --- | --- | --- | --- | --- | --- | --- | --- | --- | --- | --- | --- | --- | --- | --- |
| N individuals | 29 (all haplotypes) | Difference from best | P | 16 | Difference from best | P | 14 | Difference from best | P | 16 | Difference from best | P | 14 | Difference from best | P |
| Constraint | -LnL |  |  | -LnL |  |  | -LnL |  |  | -LnL |  |  | -LnL |  |  |
| None (best tree) | 3844.88902 | best |  | 2898.55180 | best |  | 820.97865 | best |  | 1347.34941 | best |  | 827.57482 | Best |  |
| *G. f. fuscipes* | 3860.53471 | 15.64569 | 0.139 | 2914.07150 | 15.51970 | 0.069 | 836.16551 | 15.18686 | **0.029** | 1359.50187 | 12.15246 | 0.199 | 827.57482 | 0.00000 | =best |
| *G. f. martinii* | 3844.88902 | 0.00000 | =best | 2898.55180 | 0.00000 | =best | 820.97865 | 0.00000 | =best | 1347.34941 | 0.00000 | =best | ND | ND | ND |
| *G. f. quanzensis* | 3886.86183 | 41.97281 | **0.003** | 2899.27982 | 0.72802 | 0.729 | 820.97865 | 0.00000 | =best | 1353.32764 | 5.97823 | 0.466 | 827.57482 | 0.00000 | =best |
| Ethiopia | 3844.88902 | 0.00000 | =best | 2898.55180 | 0.00000 | =best | 820.97865 | 0.00000 | =best | 1347.34941 | 0.00000 | =best | ND | ND | ND |

**Footnote:** Rejection of the hypothesis occurs when P</=0.05. For the mtDNA data set the 16 individuals that were sequenced at all loci were analysed together with and then separately from the remaining specimens for which only mtDNA data were available. This was in order to compare similar sized data sets for mitochondrial, symbiont and nuclear DNA data sets. The discrepancy between the sample size for *ITS1* and *Wigglesworthia YcfW* loci (n=14) and the *Period* and *COI+ND2* loci (n=16) is due to the lack of unique Bena Tschibangu and Buvuma genotypes from the former.

ND: not determined
