# Supplementary material for: Cryptic Diversity within the Major Trypanosomiasis Vector Glossina fuscipes Revealed by Molecular Markers
Source: PLoS Negl Trop Dis. 2011 Aug 9;5(8):e1266. doi: 10.1371/journal.pntd.0001266 (PMC3153427; doi:10.1371/journal.pntd.0001266)
Supplement: Table S4 — FIS per microsatellite locus and over all loci for each population. (DOC) [file pntd.0001266.s010.doc]

Table S4. F_IS_ per microsatellite locus and over all loci for each population.

| Population | Locus | | | | | F_IS_ |
| --- | --- | --- | --- | --- | --- | --- |
|  | GfA3 | A10 | GfB8 | GfB101 | GfB105 |  |
| Kinshasa | 0.249 | **0.943** | 0.586 | 0.099 | 0.121 | **0.423** |
| Kisantu | 0.006 | 0.765 | **1** | 0.444 | 0.213 | **0.51** |
| Madimba | -0.058 | **0.867** | **0.702** | 0.297 | **0.444** | **0.413** |
| Ethiopia | 0.046 | **0.914** | 0.292 | 0.053 | -0.013 | **0.391** |
| Ungoye | 0.102 | 0.625 | **0.714** | 0.13 | **0.588** | **0.491** |
| Manga | -0.061 | 1 | **0.49** | -0.039 | **0.869** | **0.439** |
| Rusinga | 0.086 | **0.824** | **0.706** | -0.196 | **0.77** | **0.489** |
| Bunghazi | -0.138 | **0.762** | **0.617** | 0.359 | 0.397 | **0.405** |
| Busime | -0.007 | 0.351 | 0.381 | 0.109 | 0.327 | **0.275** |
| Buvuma | -0.008 | **0.547** | **0.652** | 0.012 | -0.013 | **0.181** |
| Kigoma | 0.29 | -0.023 | NA | 0.006 | 0.195 | 0.135 |
| Bena Tschibangu | **0.32** | 0.122 | **0.7** | 0.197 | **0.714** | **0.451** |
| Moyo | 0.132 | NA | **0.576** | 0.167 | **0.61** | **0.365** |

Footnote: Text highlighted in **bold type** indicates significant F_IS_ values (corrected for multiple testing)
