# Supplementary material for: Cryptic Diversity within the Major Trypanosomiasis Vector Glossina fuscipes Revealed by Molecular Markers
Source: PLoS Negl Trop Dis. 2011 Aug 9;5(8):e1266. doi: 10.1371/journal.pntd.0001266 (PMC3153427; doi:10.1371/journal.pntd.0001266)
Supplement: Table S5 — Estimated null allele frequencies at each microsatellite locus and population inbreeding coefficient F for each population. (DOC) [file pntd.0001266.s011.doc]

Table S5: Estimated null allele frequencies at each microsatellite locus and population inbreeding coefficient F for each population.

| Population |  | | | | | F (PIM) |
| --- | --- | --- | --- | --- | --- | --- |
|  | GfA3 | A10 | GfB8 | GfB101 | GfB105 |  |
| Kinshasa | 0.10 | 0.39 | 0.23 | 0.04 | 0.03 | 0.089 |
| Kisantu | 0.00 | 0.24 | 0.46 | 0.19 | 0.05 | -1 |
| Madimba | 0.00 | 0.30 | 0.32 | 0.09 | 0.19 | 0 |
| Ethiopia | 0.00 | 0.40 | 0.10 | 0.02 | 0.00 | 0 |
| Ungoye | 0.02 | 0.23 | 0.28 | 0.05 | 0.23 | 0.157 |
| Manga | 0.00 | 0.29 | 0.22 | 0.04 | 0.34 | -1 |
| Rusinga | 0.00 | 0.33 | 0.30 | 0.00 | 0.31 | 0 |
| Bunghazi | 0.00 | 0.29 | 0.27 | 0.16 | 0.13 | 0 |
| Busime | 0.00 | 0.14 | 0.16 | 0.00 | 0.12 | 0 |
| Buvuma | 0.00 | 0.20 | 0.23 | 0.00 | 0.02 | 0 |
| Kigoma | 0.10 | 0.00 | NA | 0.00 | 0.05 | 0 |
| Bena Tschibangu | 0.15 | 0.07 | 0.31 | 0.05 | 0.24 | 0.389 |
| Moyo | 0.05 | 0.00 | 0.21 | 0.09 | 0.23 | -1 |

Footnote: Dark shading indicates frequency ≥ 0.3, light shading indicates 0.1 ≤frequency < 0.3.

Null allele estimates were generated using the EM algorithm (Dempster *et al* 1977. *Journal of the Royal Statistical Society Series B- Methodological* **39**, pp 1-38).

F (PIM) – inbreeding coefficient F over all loci estimated in parallel with null allele frequency using the population inbreeding model (Chybicki and Burczyk 2009. *Journal of Heredity* **100**, pp106-113).
