# Supplementary material for: Cryptic Diversity within the Major Trypanosomiasis Vector Glossina fuscipes Revealed by Molecular Markers
Source: PLoS Negl Trop Dis. 2011 Aug 9;5(8):e1266. doi: 10.1371/journal.pntd.0001266 (PMC3153427; doi:10.1371/journal.pntd.0001266)
Supplement: Text S1 — Methods for the ITS1 based species “diagnostic” and period gene sequencing. (DOC) [file pntd.0001266.s012.doc]

*ITS1* based subspecies ‘diagnostic’

Using the ITS1 alignment (Figure S1) Primers were designed such that the 3’ nucleotide was an exact match to one or two species, and a mismatch to the other one or two. The penultimate nucleotide was a mismatch to all the species. This further destabilized primer: template duplex formation if the last position was a mismatch, but permitted duplex formation and amplification if the last position was a match. This strategy has previously been reported to improve the specificity of SNP identification and species ID PCRs (Wilkins *et al*, 2006. *Malaria Journal* **5**, e125). PCR conditions: a universal reverse primer: ITS1-univ_R: CCTGCGGAAGGATCATTAAA at 0.83µM and three forwards primers: mar-quan_MIS: CCGTCAAAATCCCTTTTATATATTTAGG at 0.83µM, mar-MIS: GTCTTAAGGTTCATTTTGTTAAAATTAG at 0.83µM and fus-quan_MIS: GCCCATTTATTTTGGACTCC at 0.21µM were combined with reaction buffer (final concentration 1.56mM MgCl_2_), 0.8 units of Kapa Taq and 0.83mM dNTPs. 35 amplification cycles of 95°C for 30 seconds, 59°C for 30 seconds and 72°C for 30 seconds were used.

*Period* gene sequencing.

Sequencing of 3’ regions of the *period* gene for the 2070bp alignment: primer pairs GlossPerfor2 (CTCGCATAAGCATCCCAAAT) and GlossPerrev2 (CGAAATCTCTTTGCATTTCCA), GlossPerfor3 (TCATATGGAAATGCAAAGAGATTT) and GlossPerrev3 (GCTCCTTGCTGATTTTTCGT) Permiddegenfor2 (TTCTGYAGCAARCCCTATCG) and Permiddegenrev2 (ARCCYCCYCTTTTGATCC). PCR products were sequenced using the same primers used in the amplification, exceptGlossPerfor3-Gloss Perrev3 which was additionally sequenced using the internal sequencing primer Perrev4 (AATCAGTTTGTTGCGGAGGT). PCR conditions: 25μl reactions contained 1μl template, 0.8mM dNTP, MgCl_2_ as shown in the table below, each primer at 0.5μM and 0.08 microlitres (0.4 units) Kapa Taq polymerase. 35 amplification cycles were performed at the temperatures shown in the supplementary table 2. On the basis of comparison of *G. fuscipes* 2070bp sequences, the 5’ region was the richest in SNPs and was selected for amplification to make a *G. fuscipes* phylogeny.
